# Efficacy of virtual reality for pain relief in medical procedures: a systematic review and meta-analysis

**DOI:** 10.1186/s12916-024-03266-6

**Published:** 2024-02-14

**Authors:** Jhia J. Teh, Dominic J. Pascoe, Safiya Hafeji, Rohini Parchure, Adam Koczoski, Michael P. Rimmer, Khalid S. Khan, Bassel H. Al Wattar

**Affiliations:** 1 Department of Metabolism, Digestion and Reproduction, Faculty of Medicine, Imperial College London, London, UK; 2 School of Medicine, University of St Andrews, St Andrews, UK; 3 Kings College Hospital, Denmark Hill, Brixton, London, UK; 4 University College London, London, UK; 5 MRC Centre for Reproductive Health, Institute of Regeneration and Repair, Edinburgh BioQuarter, University of Edinburgh, Edinburgh, UK; 6. Johns Hospital, Livingston, West Lothian Scotland, UK; 7 Department of Preventive Medicine and Public Health, University of Granada, 18071 Granada, Spain; 8 CIBER Epidemiología y Salud Pública (CIBERESP), Instituto de Salud Carlos III (ISCIII), Madrid, Spain; 9 Beginnings Assisted Conception Unit, Epsom and St Helier University Hospitals, London, UK

**Keywords:** Analgesia, Pain, Systematic review, Virtual reality

## Abstract

**Background:**

Effective pain control is crucial to optimise the success of medical procedures. Immersive virtual reality (VR) technology could offer an effective non-invasive, non-pharmacological option to distract patients and reduce their experience of pain. We aimed to evaluate the efficacy of Immersive virtual reality (VR) technology in reducing patient’s pain perception during various medical procedures by conducting a systematic review and meta-analysis.

**Methods:**

We searched MEDLINE, EMBASE, CENTRAL, CINAHL, and SIGLE until December 2022 for all randomised clinical trials (RCT) evaluating any type of VR in patients undergoing any medical procedure. We conducted a random effect meta-analysis summarising standardised mean differences (SMD) with 95% confidence intervals (CI). We evaluated heterogeneity using *I* ^2^ and explored it using subgroup and meta-regression analyses.

**Results:**

In total, we included 92 RCTs (*n* = 7133 participants). There was a significant reduction in pain scores with VR across all medical procedures (*n* = 83, SMD − 0.78, 95% CI − 1.00 to − 0.57, *I* ^2^ = 93%, *p* = < 0.01). Subgroup analysis showed varied reduction in pain scores across trial designs [crossover (*n* = 13, SMD − 0.86, 95% CI − 1.23 to − 0.49, *I* ^2^ = 72%, *p* = < 0.01) vs parallel RCTs (*n* = 70, SMD − 0.77, 95% CI − 1.01 to − 0.52, *I* ^2^ = 90%, *p* = < 0.01)]; participant age groups [paediatric (*n* = 43, SMD − 0.91, 95% CI − 1.26 to − 0.56, *I* ^2^ = 87%, *p* = < 0.01) vs adults (*n* = 40, SMD − 0.66, 95% CI − 0.94 to − 0.39, *I* ^2^ = 89%, *p* = < 0.01)] or procedures [venepuncture (*n* = 32, SMD − 0.99, 95% CI − 1.52 to − 0.46, *I* ^2^ = 90%, *p* = < 0.01) vs childbirth (*n* = 7, SMD − 0.99, 95% CI − 1.59 to − 0.38, *I* ^2^ = 88%, *p* = < 0.01) vs minimally invasive medical procedures (*n* = 25, SMD − 0.51, 95% CI − 0.79 to − 0.23, *I* ^2^ = 85%, *p* = < 0.01) vs dressing changes in burn patients (*n* = 19, SMD − 0.8, 95% CI − 1.16 to − 0.45, *I* ^2^ = 87%, *p* = < 0.01)]. We explored heterogeneity using meta-regression which showed no significant impact of different covariates including crossover trials (*p* = 0.53), minimally invasive procedures (*p* = 0.37), and among paediatric participants (*p* = 0.27). Cumulative meta-analysis showed no change in overall effect estimates with the additional RCTs since 2018.

**Conclusions:**

Immersive VR technology offers effective pain control across various medical procedures, albeit statistical heterogeneity. Further research is needed to inform the safe adoption of this technology across different medical disciplines.

**Supplementary Information:**

The online version contains supplementary material available at 10.1186/s12916-024-03266-6.

## Background

Pain is the commonest symptom encountered in clinical practice often manifesting as an unavoidable consequence of medical procedures. Effective pain management is crucial to optimise medical procedures, boost patients’ satisfaction [1–3], reduce their anxiety, reduce hospital stay and minimise long-term analgesic dependence [4–6]. The use of immersive virtual reality (VR) technology has emerged as a potential tool to distract patients and to modify their perception of pain. Its adoption in clinical practice remains limited.

The search for effective, safe, and cheap analgesic treatment options is a priority accelerated in part by the emerging opiates epidemic in several countries associated with dependence risk and narrow safety profile [7, 8]. VR technology seems to offer a credible option for effective acute pain relief either as an alternative or as a combined treatment as part of a multi-modal pain relief strategy [9].

The term ‘virtual reality’ was coined by Jaron Lanier, a writer, musician, visual artist, and computer scientist, who first used it in 1986. The first application of VR in healthcare dates back to the beginning of the 1990s. It stemmed from the need to visualize complex medical data, especially when planning surgical treatment [10]. Since then, the use of VR technology in medicine proliferated into several domains including surgical training, neuropsychiatry, acute and chronic pain management, and rehabilitation [10, 11].

VR devices are designed to alter one’s perception of presence in an alternate reality and augment their immersion, and interactivity [12]. Today, several cheap and user-friendly devices offer an immersive environment largely delivered via high-resolution head-mounted displays (HMDs) with built-in sound capabilities [13]. In clinical practice, immersive VR experience aims to distract patients during medical procedures, suppressing their appreciation of immediate physical surroundings, allowing them to escape into an alternative reality away from the painful stimuli [14–16]. Early VR equipment had several technological barriers that limited their use in everyday practice, including high cost, relatively large size, complex operating interface, and user unfamiliarity [17]. Recent advances in audio-visual technology, driven by the wide use of smartphones, have enabled the development of affordable and user-friendly equipment [18]. Coupled with bespoke medical software, these new VR devices offer patients a versatile immersive visual and auditory experience that could be adopted across different clinical settings [11, 19].

Several meta-analyses have evaluated the efficacy of VR showing a beneficial effect with its use. Georgescu et al. [20] performed a meta-analysis for randomised trials that evaluated VR until 2018 (*n* = 27 RCTs, 1452 patients) showing a beneficial effect for pain reduction following medical procedure although the findings were limited by high heterogeneity and high trial risk of bias [20]. Scapin et al. [21] performed a systematic review including [22] randomised trials on the use of VR in burn patients. The findings were also supportive of the role of VR as an effective complementary drug strategy for pain relief in burn patients [21]. However, these reviews were either limited to specific clinical situations, suffered from high heterogeneity, or lacked detailed subgroup analyses to explore the reasons for heterogeneity [21].

In the year 2022, there have been 24 new randomised clinical trials (RCT) [22–45] published evaluating VR technology highlighting the increased interest in this technology and offering further insight into its applicability across different medical disciplines. Still, the translation of this evidence has remained poor with respect to implementation of VR technology at scale and with variation in practice where medical specialities have taken steps towards adoption. Appreciation of the role of VR for pain relief can be aided by updated evidence synthesis [46].

In this systematic review, we conducted a comprehensive assessment of the evidence on VR efficacy as a non-invasive and non-pharmacological pain management method in patients undergoing different medical procedures. We performed an overall evidence synthesis pooling data from all relevant RCTs in addition to bespoke subgroup and meta-regression analyses to help interpret the evidence [17, 47].

## Methods

We conducted this systematic review using a prospectively registered protocol (CRD 42020195919) [48] and reported in accordance with PRISMA guidelines [49].

### Literature search

We searched major electronic databases (MEDLINE, EMBASE, Cochrance CENTRAL, CINAHL, and SIGLE) for randomised trials that evaluated the efficacy of immersive VR technology equipment for pain relief from inception until December 2022. We developed a comprehensive and inclusive search strategy using MeSH search terms and combined them using the Boolean ‘AND’ and ‘OR’ (Additional File 1: Appendix S1). We applied this search strategy to individual databases after amending it to the specification of each database. We then deduplicated the results and produced a final long list of citations. We manually searched the bibliographies of relevant studies to identify any additional trials not captured by our electronic database search. We also conducted supplementary searches in Google Scholar and Trip database to identify additional studies of relevance [50]. We did not apply any search filters or language restrictions. Relevant citations in non-English were obtained and translated for assessment against our inclusion criteria.

### Study selection

Five independent reviewers (JJT, DP, SH and RP, AK) completed the study screening and inclusion process in two stages. First, titles and abstracts were screened to identify potentially relevant studies following which, the full text of relevant articles were reviewed against our inclusion criteria. We included all randomised trials of any design that evaluated the efficacy of any immersive VR technology equipment for pain relief during any medical procedure, including labour and childbirth. We initially planned this review to include only adult participants and later extended this to include paediatric participants to provide a more comprehensive evidence synthesis. We excluded non-randomised studies, review articles, and animal studies. We also excluded studies that assessed distraction techniques only (e.g. a display screen with no immersive capabilities), studies in dental procedures, and those that did not assess pain using a standardised measurement tool or reported on pain scores more than an hour after the procedure. Discrepancies and disagreements between reviewers were discussed and resolved in consensus with two additional reviewers (MPR and BHA).

### Data extraction

Three reviewers (JJT, DP, SH, AK) extracted data in duplicate using a piloted electronic data extraction tool. We collected data on study design (crossover vs parallel), intervention settings, population characteristics, inclusion and exclusion criteria, type of VR technology and equipment used, nature of the medical procedure or intervention, loss to follow-up, and dropouts. Our primary outcome was pain scores measured immediately after or within an hour of the procedure. We also collected data on anxiety scores where relevant. In trials including paediatric patients, we included the parents’ reported pain scores.

### Assessment of risk of bias

We assessed the risk of bias in included trials in duplicate (JJT, RP, AK, DP, MPR, SH) using the Cochrane Risk of Bias assessment tool 2.0 [51]. We assessed studies in five domains: participant randomisation and sequence generation, allocation concealment, outcome assessment, completeness of outcome data, and selective outcome reporting. Due to the nature of the intervention, we did not penalise unblinded trials. Studies with a crossover design were assessed using a modified version of an established tool [52]. We assessed the risk of bias in these studies for appropriate crossover design, randomisation and order of receiving the treatment, risk of carry-over effect, data collection, allocation concealment, outcome detection, data completeness, and selective outcome reporting.

### Data synthesis

We pooled data using a meta-analysis with a random effect and adjusted using restricted maximum likelihood (REML) [53]. We reported on the difference in pain scores measured using standardised mean difference (SMD) with 95% confidence intervals (CI). We assessed any detected heterogeneity using the *I* ^2^ statistics. The *I* ^2^ index is an approach to quantify heterogeneity in meta-analyses. *I* ^2^ provides an estimate of the percentage of variability in results across studies that is due to real differences and not due to chance. The *I* ^2^ index measures the extent of heterogeneity by dividing the result of Cochran’s *Q* test and its degrees of freedom by the *Q*-value itself. An *I* ^2^ of less than 25% is usually viewed as low heterogeneity, between 25 and 50% as moderate, and over 50% as high heterogeneity.

We planned subgroup analyses to investigate potential effect modifiers (patient age group (paediatric patients defined as < 16 years old) vs adults), type of medical intervention (venepuncture-related procedures, minimally invasive medical procedures (defined as any medical procedure conducted in office setting without the need for general anaesthesia), dressing changes in burn patients, and childbirth), trial design (parallel group vs crossover trials), the trial quality as assessed using the risk of bias tool), the type of VR technology (interactive: arbitrarily defined when VR software is asking the participant to take part in specific activities compared to a passive VR experience), the VR delivery settings (inpatient vs outpatient vs emergency department) and assessed their impact on the effect estimates using a meta-regression [54]. We explored potential sources of heterogeneity using a leave-one-out analysis and a sensitivity analysis excluding potential outliers. We also investigated the risk of publication bias using Egger’s test, a funnel plot, and Galbraith plot to identify potential outliers [55]. Where publication bias was detected, we explore potential impact using the trim and fill method [56] to estimate and adjust for the number and outcomes of missing studies in the meta-analysis. We conducted a cumulative meta-analysis for selected outcomes to evaluate temporal trends and changes in effect estimate over time as new trials emerged [57]. Statistical analyses were conducted in STATA V17 (StataCorp, TX) and Open Meta-analyst software (Brown University; Providence, RI, USA).

### Patient and public involvement

No input was sought from lay service consumers in the design, conduct, and reporting of this systematic review.

## Results

**Fig. 1:**
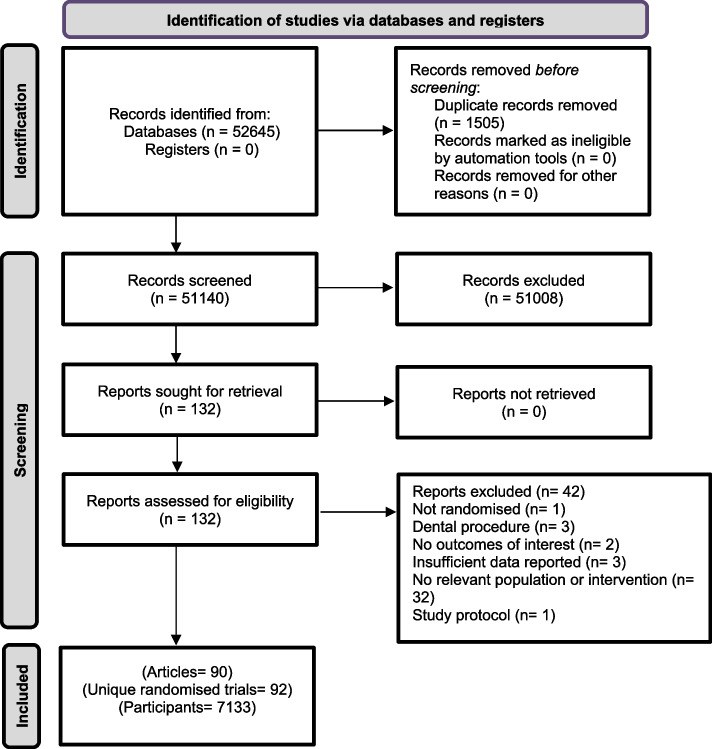
Selection and inclusion process for randomised trials evaluating the effectiveness of virtual reality for pain control in medical procedures

We identified 51,140 potentially relevant citations, of which we assessed 132 studies against our inclusion criteria and included 90 articles reporting on 92 unique RCTs in our meta-analysis (7133 participants) (Fig. 1) (Additional File 1: Appendix S2. (40 studies were excluded [58–99]). No relevant citations were identified in non-English. The majority of included RCTs had a two-group parallel design (77/92, 84%), including a three-arm RCT [100], and less than one fifth had a crossover design (15/92, 16%).

Forty-two of included trials enrolled adults only (42/92, 46%), three had a mixed population, and fifty included paediatric participants only (50/92, 54%). The majority of trials were conducted in high-income countries; twenty-seven trials were conducted in the USA (27/92, 29%) while nineteen were conducted in Turkey (19/92, 21%), seven trials in Australia, Canada China respectively (7/92, 8%) (Additional File 1: Table S1).

The type of VR technology and equipment used across included trials evolved over time from interactive, immersive games hosted on a personal computer to immersive environment experiences with user-controlled interactive interface and real-time feedback (Table 1). Trials conducted over the last 10 years evaluated newer VR technology with sound immersive augmentation (13/47, 26%) [101–113] and hand-held mobile phones or mounted goggles (27/47, 58%) [100, 102, 103, 105, 106, 110, 111, 113–131].

**Table 1:** Description of the VR equipment and software used in randomised trials evaluating the effectiveness of virtual reality for pain control in medical procedures

Study	VR equipment	VR software	Interactive or non-interactive
**Akin 2021 **[132]	VR Box 3D virtual reality glass	Video images recorded on the phone of the pregnant woman by looking at the baby’s face with the help of a 3D/4D probe	Non-interactive
**Atzori 2018 **[107]	VR helmet (HMZ T-2 3D viewer Sony) with 45° diagonal field of view supported by laptop, latex-free earphones	Interactive game (Snow World)	Interactive
**Atzori 2022 **[45]	VR helmet, the Personal 3D Viewer Sony: HMZ T-2, supported by a laptop	Interactive game (Snow World)	Interactive
**Aydin and Ozyazicioglu 2019 **[133]	VR headset	Non-interactive 3D video stimulating a submarine journey to discover things in the virtual aquarium	Non-interactive
**Basak 2021** [134]	3-D audio-visual presentation was watched using VR glasses	Submarine view video	Non-interactive
**Boonreunya 2022 **[44]	VR headset Olympus GIF-HQ190. The content on the VR screen showed nature scenarios in relaxing mode	Commercially available VR program and specific content about nature scenarios and sightseeing in relaxing mode	Non-interactive
**Bosso 2023 **[43]	Oculus GO headset with sound played through headphones with active noise reduction	Zen garden developed by Healthy Mind	Non-interactive
**Bozdoğan Yesilot 2022 **[135]	VR headset (not specified)	Relaxing video	Non-interactive
**Brunn 2022 **[41]	Oculus Go headset with guided meditation VR App	Nokia Spot 1 environment and 10-min guided Zen meditation	Interactive
**Karaveli—Cakir 2021 **[136]	Android mobile phone placed in Cardboard Super Flex Goggles	‘A walk on the beach’	Non-interactive
**Canares 2021 **[137]	Commercially available VR headset with game	Not specified	Not specified
**Carrougher 2009 **[108]	VR helmet with head-position tracking and audio feedback	Interactive game (Snow World)	Interactive
**Carus 2022 **[40]	Oculus Quest All-in-one VR Gaming Headset (128 GB) VR system	Several virtual environments, including orange sunset, green meadows, black beginning, red savannah, blue deep, blue moon, blue ocean, white winter, and red fall	Non-interactive
**Chan 2007 **[123]	Super high-resolution interactive glasses with graphic animation and multi-sensory input with sound and sight to create an immersive environment taking into account the participants’ age group, psychomotor developmental abilities, and intellectual capabilities	Interactive game (Ice Cream Factory) controlled with a PC mouse	Interactive
**Chan 2019 **[122]	Google Pixel XL/Google Daydream VR headset	Interactive game (Underwater adventure) with relaxation and progressing to marine scenes	Interactive
**Chan 2019 **[122]	Google Pixel XL/Google Daydream VR headset	Interactive game (Underwater adventure) with relaxation and progressing to marine scenes	Interactive
**Chang 2022 **[39]	Oculus Quest headset (Facebook Technologies)	SILVER software enacts a story centred on the ‘Burp’s Magic Tower’	Non-Interactive
**Chen 2020 **[119]	HMD with headphones playing videos from downloaded VR apps on iPhones	Various immersive non-interactive videos of virtual environments (roller coasters, space exploration a wildlife park and travel destinations)	Non-Interactive
**Clerc 2021 **[138]	VR headset (VOX + Z3 3D; Emerge Technologies, Coppell, Texas) and smartphone (Asus Zenfone 2 ZE551ML; ASUSTek Computer Inc., Taipei, Taiwan)	VR Roller Coaster	Non-interactive
**Das 2005 **[124]	HMD with a tracking system steered by a PC mouse	Immersive interactive game with virtual environment controlled by moving the participant’s head, neck, decoder	Interactive
**Deo 2021 **[120]	Oculus Go with a head-mounted display and built-in audio drivers	8-min video called ‘Forest of Serenity’ commissioned by St Giles Hospice, developed by Holosphere and narrated by Sir David Attenborough	Interactive
**Ding 2019 **[106]	HMD (eMagin z800) with 40° view field used with an integrated audio system and a FasTrak control box	Interactive game (Snow World 2.1)	Interactive
**Dumoulin 2019 **[121]	HMD (eMagin z800) with built-in motion tracker, steered by a wireless PC mouse	Immersive Interactive game (Health Shoot The Flies)	Interactive
**Ebrahimian 2022 **[38]	Virtual reality glasses (Samsung Gear VR Virtual Reality Headset with Samsung Mobile S7)	360° video with nature landscapes	Non-interactive
**Erdogan 2021 **[116]	VR glasses and headsets (Samsung Galaxy) powered by Samsung Galaxy Note 5 N920 smartphone	Non-interactive 3D age-appropriate animation (Dinosaur)	Non-interactive
**Estrella-Juarez 2023 **[139]	Bnext 3D glasses and 360° images	Images of the ocean floor with relaxing sounds	Non-interactive
**Fouks 2022 **[37]	Head-mounted display (SootheVR: AppliedVR, Los Angeles, California)	Immersive module of diving in a lagoon	Interactive
**Frey 2019 **[125]	Samsung Gear Oculus VR headset fitted with Samsung Galaxy S5 Note phone	Non-interactive animation Cartoon videos	Non-interactive
**Genc 2022 **[36]	VR glasses connected with smart mobile phone	Nature video scenes recorded on YouTube at 360° were watched for 10 min	Non-interactive
**Gerçeker 2018 **[126]	HMD powered by a Galaxy S7 phone with a hand control and noise-reducing headphones	Interactive environment (Ocean Rift) showing scuba diving simulation with sounds of manatee calls and breathing underwater, with relaxing music and user input via head tracking and a hand control to stimulate taking underwater photos	Interactive
**Gerçeker 2020 **[100]	Samsung Gear Oculus headset connected to Samsung Galaxy S5 Note mobile phones with audio feedback	Non-interactive video (Rollercoster or Ocean Rift)	Non-interactive
**Gerçeker 2020 **[100]	Samsung Gear Oculus headset connected to Samsung Galaxy S5 Note mobile phones with audio feedback	Non-interactive video (Rollercoster or Ocean Rift)	Non-interactive
**Gerçeker 2021 **[118]	Samsung Gear Oculus headset connected to Samsung Galaxy S7 Edge mobile phones, head tracking and hand controller	Interactive environment (Ocean Rift, Rilix VR or In the eyes of animal)	Interactive
**Goergen 2022 **[35]	Samsung® A5 mobile phone adapted to Trust Urban® Exos 3D virtual reality glasses and a headset	App-video was playing simulating a ride on rails (without sudden changes that could scare him or change his heart rate)	Non-interactive
**Gold 2006 **[112]	High-performance professional immersive HMD with multi-sensory audio and tactile feedback with inertial tracking and music via headphones	Interactive game (Street Luge)	Interactive
**Gold 2018 **[127]	Age-appropriate VR goggle (Samsung Galaxy G6 mobile-based Gear VR goggles or Google Pixel mobile-based Merge VR goggles) multi-sensory (visual and auditory) immersive system and head-tracking sensors	Interactive game (Bear Blast)	Interactive
**Gold 2021 **[140]	Samsung Gear VR [Samsung Electronics] or Merge VR [Merge Labs], VR game Bear Blast; AppliedVR	VR game Bear Blast	Interactive
**Goldman 2021a **[141]	VR Headset (ReTrak Utopia 360 VR Headset) and smartphone (Asus Zenfone 2 ZE551ML) pre-loaded with a VR Roller Coaster app (VR Roller Coaster, Frag)	Pre-loaded with a VR Roller Coaster app (VR Roller Coaster, Frag)	Interactive
**Goldman 2021b **[142]	VOX + Z3 3D Virtual Reality Headset (China), an Asus Zenfone 2 ZE551ML mobile de- vice (Taiwan)	Pre-loaded with a VR Roller Coaster app (VR Roller Coaster, Frag)	Interactive
**Gray 2021 **[117]	Oculus Go VR goggles and a hand-held controller	VR game called SpaceBurgers	Interactive
**Guo 2015 **[128]	Ultra-high-resolution 3D glasses, headphones, and a PC mouse	Non-interactive videos (Afanda)	Non-interactive
**Hoffman 2001 **[131]	VR helmet with motion-sensing system. Circular eyepiece with a 60° diagonal field of view per eye with PC keyboard controls motion-sensing system to complete dedicated tasks (e.g. pick up virtual objects, tactile augmentation)	Interactive game (Snow World) offering Immersive 3D interactive computer-simulated environment	Interactive
**Hoffman 2008 **[109]	VR helmet with a Microsoft SideWinder joystick and audio effects	Interactive game (Snow World)	Interactive
**Hoffman 2019 **[105]	Water-friendly VR goggles (MX90) held by robot-like articulated arm goggle holder, with battery-powered laptop and audio-visual unit, steered by a wireless computer mouse	Interactive game (Snow World)	Interactive
**Hsu 2022 **[34]	VR headset HTC Vive (HMD)	VR Cosmos fully immersive view with the wireless hand controller mirrored the participants’ hand actions	Interactive
**Hua 2015 **[143]	HMD with laptop joystick	Chinese version of the Ice Age 2: The Meltdown game (Twentieth Century Fox, Sierra Entertainment)	Interactive
**Huang 2020 **[115]	Samsung Gear VR HMD or Oculus Rift Development Kit 2 HMD with noise-cancelling headphone	Custom-designed version of the software ‘Iceland’ and Snow World	Non-interactive
**Hundert 2021 **[33]	VR head-mounted display, noise-cancelling headphones (to deliver sound) and held a wireless Bluetooth controller	Auditory and visual stimuli (a game which consisted of aiming rainbow balls at sea creatures as they explored an underwater environment in search of treasure)	Interactive
**Jahani Shoorab 2015 **[110]	VR glasses playing 3D film with two external headphones (3D blue-ray/DVD player full HD)	Non-interactive videos (Dolphin and Whales)	Non-interactive
**Jeffs 2014 **[144]	VR helmet mounted on articulated arm tripod device with Bose Quiet comfort 3 headphones and trackball controller	Interactive game (Snow World)	Interactive
**Joo 2021 **[145]	Samsung Gear HMD compatible with the Android platform operating on a Galaxy 7.0 device	3D VR software consists of a seashore view with Korean language narrations designed to induce relaxation (NUVO program by Oncomfort SA, Wavre, Belgium)	Non-interactive
**Karaman 2021 **[146]	G3 smartphone, the 3D VR Player (GO VR) application and VR Box Glasses	‘A walk on the beach’	Non-interactive
**Kaya 2022 **[32]	Samsung Gear and Oculus Rift VR headsets	‘Merry Snowballs VR’ application	Interactive
**Ketsuwan 2022 **[31]	HMD (BOBOVR Z6 Wireless Bluetooth version; Winhoo, Guangdong, China) in combination with an Apple iPhone 12 smartphone	Landscape image of a snowy mountain	Non-interactive
**Kipping 2012 **[147]	HMD 3D Visor with head tracking and joystick hand controllers	Interactive game (Chicken Little/Need for Speed)	Interactive
**Konstantatos 2009 **[113]	Goggles fitted on head via a circumferential strap, disposable earpiece, commentary plus DVD footage	Non-interactive video based on hypnotherapy therapy (virtual reality relaxation)	Non-interactive
**Leopold 2022 **[30]	Unspecified	Unspecified	Non-interactive
**Litwin 2021 **[114]	Samsung GearVR head-mounted device	Child travelling underwater through the ocean (kindVR Aqua, Alameda, CA) to launch rainbow-coloured balls at nearby fish	Interactive
**Liu 2021 **[148]	Oculus Go VR goggles (head mount display hardware; hand trackers; built-in surround sound spatial audio speakers) and a hand-held controller,	VR game called SpaceBurgers	Interactive
**Liu 2022 **[29]	Head-mounted VR display (Nibiru 3.50.005)	Short clips (with a total length 30 min) featuring tropical islands and forests with soothing music: ‘With an orchid’ by Yanni	Non-interactive
**Łuczak 2021 **[149]	VR set consisting of goggles and headphones	3D world generated through the projection of images, the emission of sounds and the production of other stimuli	Non-interactive
**Luo 2023 **[28]	VR glasses (Unity3D 2018.3.10f1, Shu Rui Medical)	Rural scenery close to nature, including blue sky, floating white clouds, trees swaying in the wind, flowing water, birds in flight, and soft music	Non-interactive
**Maani 2011 **[129]	3D VR goggles with Robot-like arm, sound effects and a mouse controller	Interactive game (Snow World) controlled with a PC mouse	Interactive
**McSherry 2018 **[130]	3D VR goggles with sound effects, noise-cancelling earphones with PC mouse	Interactive game (Snow World)	Interactive
**Melcer 2021 **[150]	Oculus Go standalone VR headset	5–15-min clip showing views of rolling hills, sail boats, a tropical beach, a beautiful desert landscape and the undersea world	Non-interactive
**Momenyan 2021 **[151]	Android application was developed using the Google VR SDK, patient's head movements tracked using Samsung S3’s inertial measurement unit (IMU) sensor	360° video of nature containing beach and peaceful landscape along with the sound of nature	Non-interactive
**Osmanlliu 2021 **[152]	Oculus Rift head-mounted device	Videogame Dreamland®, developed by Oniric Interactive in collaboration with the Université du Québec en Abitibi-Témis-camingue, a classic ‘point & shoot’ game that uses head movements for aiming and a hand trigger for shooting balloons	Non-interactive
**Özkan 2020 **[101]	VR goggle compatible with iPhone 6 playing videos with sounds	Various non-interactive video	Non-interactive
**Özsoy 2022 **[153]	Preo My VR box head-mounted device	Cartoon on tablet distraction	Non-interactive
**Pathoulas 2022 **[154]	Single-use disposable VR devices with a mobile phone	Moving 3D graphics	Non-interactive
**Perdue 2022 **[22]	Oculus Go head-mounted device	Playing Ocean Rift	Interactive
**Piskorz 2020 **[58]	Head-mounted displays (Samsung gear)	Memorize blinking elements and search for them among other moving objects	Interactive
**Pratiw 2017 **[155]	VR headset with Smartphone Lenovo K4 Note	Non-interactive videos (Festivo) offering VR distraction sequence scenery like river, beach, waterfall, and lake allowing users to glide through a with 360° video	Non-interactive
**Ryu 2022 **[156]	Oculus Go head-mounted VR display	Characters of ‘Hello Carbot’ (Choi-Rock Contents Factory, Seoul, South Korea),	Non-interactive
**Sander Windt 2002 **[111]	VR glasses and earphones	Non-interactive video (Escape) offering multidimensional sight and sound experience with several videos and music in stereo sound	Non-interactive
**Schlechter 2021 **[157]	VR headsets, eye mask, iPhone (Apple Inc., Cupertino, CA) with VR software, and optional headphones	Game of narwhal swims through the ocean	Interactive
**Schmitt 2011 **[158]	nVisor SX (NVIS Inc., Reston VA), the VR-1280 (Virtual Research Systems, Aptos CA), the ProView XL 50 (Kaiser Electro-Optics, Carlsbad CA), and the ProView SR 80 and Polhemus Fastrak (Polhemus, Colchester VT) motion-sensing system (six degrees of freedom)	Interactive game (Snow World)	Interactive
**Semerci 2020** [102]	PiranhaTM VR system and headset connects to an iPhone 6 mobile phones and allows for watching and listening to VR videos	Rollercoaster video in which a rollercoaster speeds up and slows down in the forest accompanied by slow music	Non-interactive
**Smith 2020 **[159]	HMD with a Samsung Galaxy S8 smartphone, head tracking, hand controllers and touchpad	Interactive game (Skylight) with relaxing background music is also played to provide auditory stimulation	Interactive
**Soltani 2018 **[160]	VR goggles (MX90) steered by a computer mouse	Interactive game (Gliding through an icy 3D canyon)	Interactive
**Stunden 2021 **[161]	MERGE VR headset (Merge Labs Inc)	Tutorial included a dinosaur in outer space that taught the user how to interact with the elements, see in 360°, and interact with hotspots (referred to as teleportation devices)	Non-interactive
**Thybo 2022 **[26]	Oculus Go VR goggles (Khora Virtual Reality Denmark)	VR game named Freddy-the-Frog, a 3D interactive game made in cooperation with a professional VR company (Khora Virtual Reality Denmark, Copenhagen, Denmark)	Interactive
**Top 2021 **[162]	VR BOX 3.0	Video (3D Aquarium) by virtual reality glasses	Non-interactive
**Walker 2014 **[163]	VR helmet and trackball hand controller	Interactive game (Snow World)	Interactive
**Walther-Larsen 2019 **[164]	Samsung S6 mobile-based Gear VR goggles steered by a controller	Interactive 3D game (Seagull Splash)	Interactive
**Wang 2022 **[165]	Pico-G2-4KS all-in-one machine	‘Chicken Run’ puzzle game	Interactive
**Wolitzky 2005 **[166]	HMD and joystick connected to a computer	Interactive game (Virtual Gorilla) offering educational supplement for children visiting the gorilla habitat at Zoo	Interactive
**Wong 2021a **[167]	Google cardboard goggles fitted to Apple and Samsung smartphones	VR cartoons	Non-interactive
**Wong 2021b **[103]	VR goggle with imagery and auditory guidance	Non-interactive videos of a blossoming tree, ocean waves, and crackling campfire with meditative auditory guidance	Non-interactive
**Xiang 2021 **[168]	Lightweight, low-cost VR paired with an Apple iPhone 6 and detachable earphones	VR game titled Virtual River Cruise	Interactive
**Xie 2022 **[24]	3D SpaceMax software	The real scene shooting in the delivery room is added to the system to bring the 3D interactive virtual scene to life, including characters, sites, objects, environments, time and voices	Non-interactive
**Yildirim 2023 **[23]	Immersive experiments with VR glasses (Oculus Rift VR and Samsung Galaxy S7 mobile phone and headset)	3 virtual environments (i.e. roller coaster, mine craft, ocean rift)	Non-interactive

*HMD* head-mounted display, *VR* virtual reality, *PC* personal computer

## Risk of bias

For parallel-group RCTs, the overall quality of the included studies was moderate with the majority of studies showing low or moderate risk of bias for selective reporting (73/77, 95%), outcome assessment (72/77, 94%), completeness of data (74/77, 91%) and randomisation risk of bias (70/77, 91%). Still, nine trials showed high risk for adherence to intervention groups (9/77, 12%), and none reported blinding participants or assessors (Additional File 1: Figure S1). The majority of crossover trials showed a high or unclear risk of bias, specifically for carry-over effect (13/15, 87%), completeness of data (6/15, 40%) and detection bias (12/15, 80%). The risk of bias for allocation concealment was deemed to be high in ten crossover trials (10/15, 67%) (Additional File 1: Figure S1).

## Outcomes

### Pain

**Fig. 2:**
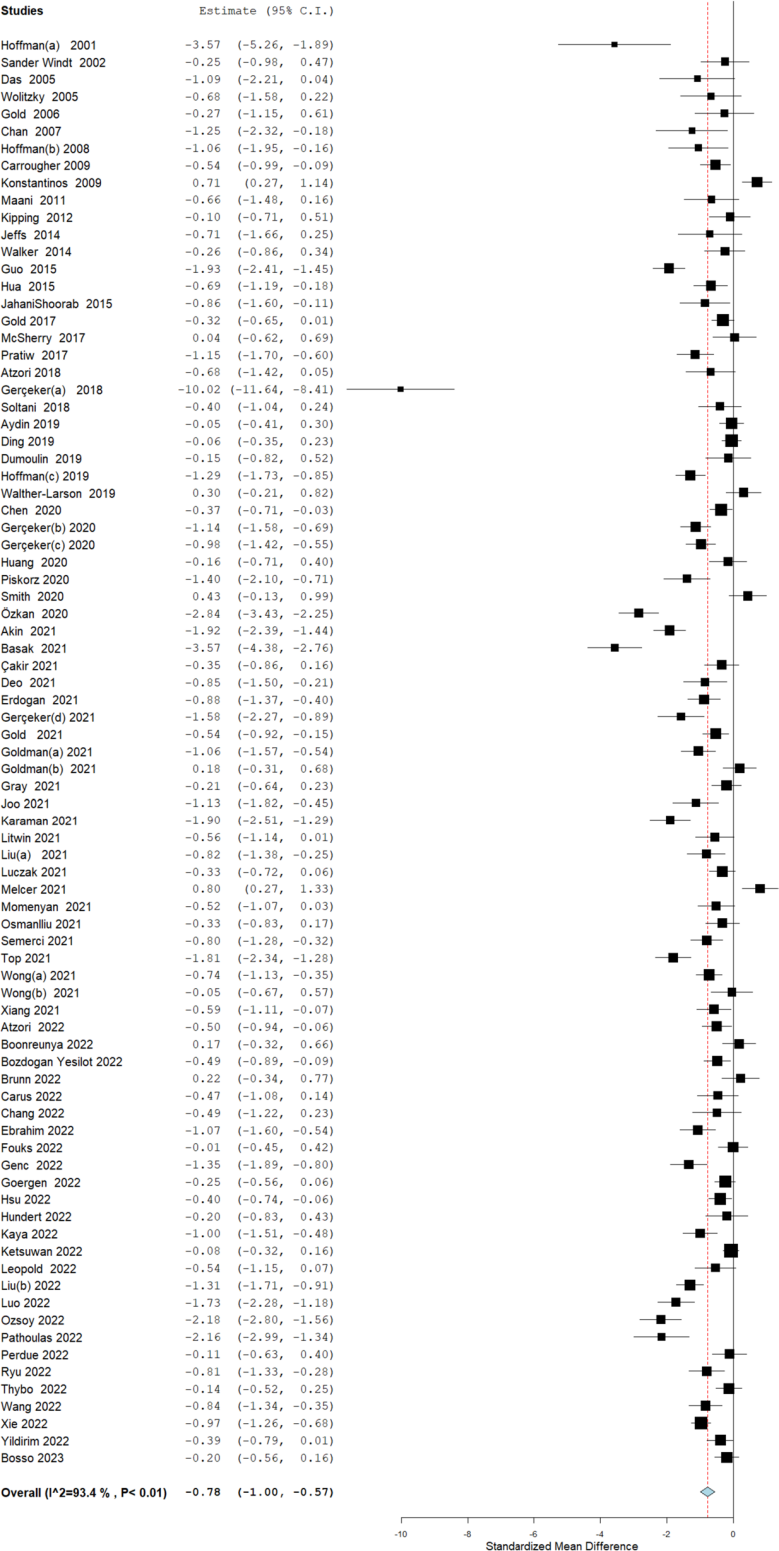
Meta-analysis on the effectiveness of VR technology for pain control compared to routine care across different medical procedures

**Fig. 3:**
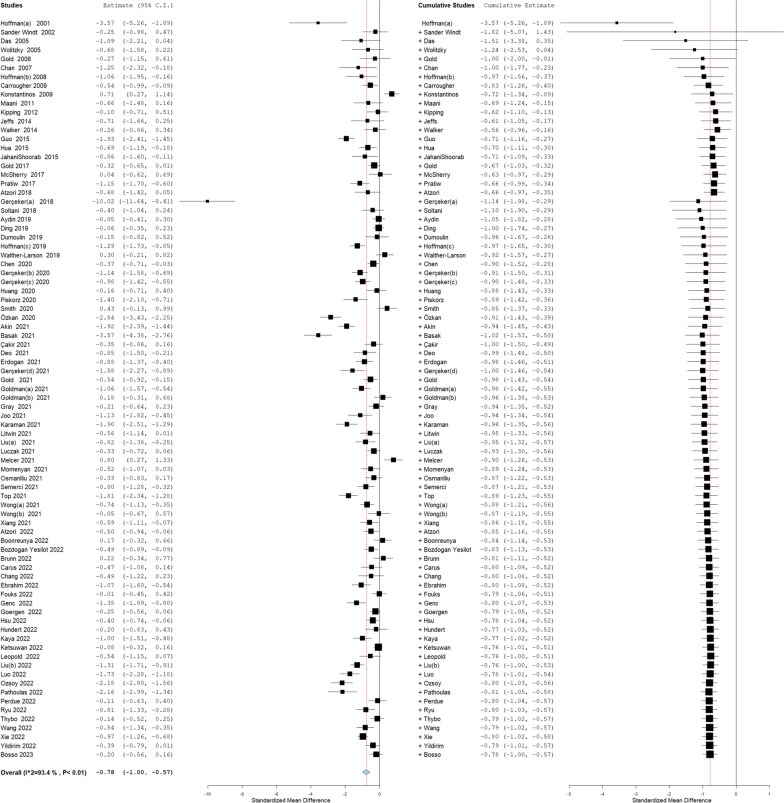
Cumulative meta-analysis on the effectiveness of VR technology for pain control compared to routine care across different medical procedures

We pooled data from 83 RCTs that reported on pain scores following any medical procedure with nine RCTs excluded from the meta-analysis due to limited outcome reporting ((Additional File 1: Appendix S3). Our meta-analysis showed a significant reduction in pain scores with the use of VR across all types of medical procedures (*n* = 83, SMD − 0.78, 95% CI − 1.00 to − 0.57, *p* = < 0.01), although heterogeneity was high (*I* ^2^ = 93%) (Fig. 2). We explored the heterogeneity using meta-regression which showed no significant effect of different covariates, including crossover trials (*p* = 0.53), minimally invasive procedures (*p* = 0.37) or among paediatric participants (*p* = 0.27). (Additional File 1: Table S2). We conducted a cumulative meta-analysis to illustrate the chronological change in the effect size of VR on reducing pain which showed no change in overall effect estimates with the addition of new RCTs since 2018 (Fig. 3). We also calculated the predictive intervals of the pooled effect estimated which shows that 95% of the true effect size falls between − 4.02 and 1.05 for all comparable populations.

We performed subgroup analyses across these three identified categories (trial design, type of medical procedure and participant age group). The reduction in pain scores was consistent across crossover trials (*n* = 13, SMD − 0.86, 95% CI − 1.23 to − 0.49, *I* ^2^ = 72%, *p* = < 0.01) and parallel-group trials (*n* = 70, SMD − 0.77, 95% CI − 1.01 to − 0.52, *I* ^2^ = 90%, *p* = < 0.01) (Additional File 1: Figure S2). Similarly, VR reduced pain across the different participant age groups, though the effect was higher in paediatric participants (*n* = 43, SMD − 0.91, 95% CI − 1.26 to − 0.56, *I* ^2^ = 87%, *p* = < 0.01) compared to adults (*n* = 40, SMD − 0.66, 95% CI − 0.94 to − 0.39, *I* ^2^ = 89%, *p* = < 0.01) (Additional File 1: Figure S2). The efficacy of VR in reducing pain was significant in participants undergoing venepuncture-related procedures (*n* = 32, SMD − 0.99, 95% CI − 1.52 to − 0.46, *I* ^2^ = 90%, *p* = < 0.01), minimally invasive medical procedures (*n* = 25, SMD − 0.51, 95% CI − 0.79 to − 0.23, *I* ^2^ = 85%, *p* = < 0.01.), dressing changes in burn patients (*n* = 19, SMD − 0.8, 95% CI − 1.16 to − 0.45, *I* ^2^ = 87%, *p* = < 0.01) and during childbirth (*n* = 7, SMD − 0.99, 95% CI − 1.59 to − 0.38, *I* ^2^ = 88%, *p* = < 0.01) (Additional File 1: Figure S2). The use of interactive VR technology did not yield significant difference (SMD − 0.72, 95% CI − 1.11 to − 0.34, *p* = 0.00) compared to using non-interactive software (SMD − 0.78, 95% CI − 0.99 to − 0.57, *p* = 0.00). VR was effective in reducing pain across different care settings including inpatient (SMD − 0.79, 95% CI − 1.02 to − 0.57, *p* = 0.00), outpatient (SMD − 0.87, 95% CI − 1.45 to − 0.28, *p* = 0.28), and emergency department (SMD − 0.80, 95% CI − 1.70 to 0.11, *p* = 0.00).

We assessed publication bias using Egger’s test, which was significant (*p* = 0.11). We visually inspected the variance in effect estimates for potential small study effect using a funnel plot (Additional File 1: Figure S3) and a Galbraith plot (Additional File 1: Figure S3) which identified several outliers although the overall precision in the effect estimate was high. We explored the potential impact of publication bias using the trim and fill method which did not identify any missing studies (Hedge’s *g* 0.00, 95%CI − 0.051 to 0.051) (Additional File 1: Figure S3).

We conducted a leave-one-out analysis, which identified five studies as potential outliers [101, 126, 131, 134, 153, 154]. We then conducted a sensitivity analysis excluding these trials, which led to a small reduction in the overall effect estimate (SMD − 0.58, 95% CI − 0.71 to − 0.45), but did not resolve the observed heterogeneity (*I* ^2^ = 82%).

### Anxiety

Thirty-one trials reported on changes in anxiety between the VR group and routine care, mainly involving minor medical procedures and venepuncture procedures [101, 111, 117, 120, 127, 128, 142]. The overall effect estimate showed a significant reduction in anxiety scores with the use of VR across all populations, although heterogeneity was high (*n* = 31, SMD − 0.82, 95% CI − 1.09 to − 0.54, *I* ^2^ = 91%, *p* = < 0.01) (Additional File 1: Figure S4). The cumulative meta-analysis showed more precise effect estimates with the addition of newer trials over the last 2 years, although the confidence interval remained relatively wide (Additional File 1: Figure S4).

We performed subgroup analyses across these three identified categories (trial design, type of medical procedure, and participant age group). The effect of VR technology on anxiety reduction was higher among paediatric participants (*n* = 15, SMD − 0.96, 95% CI − 1.37 to − 0.54, *I* ^2^ = 91%, *p* = < 0.01) compared to adults (*n* = 16, SMD − 0.68, 95% CI − 1.04 to − 0.32, *I* ^2^ = 91%, *p* = < 0.01) (Additional File 1: Figure S5). Reduction in anxiety was highest among trials that evaluated venepuncture-related procedures (*n* = 15, SMD − 0.99, 95% CI − 1.39 to − 0.58, *I* ^2^ = 90%, *p* = < 0.01) followed by minor medical procedures (*n* = 10, SMD − 0.42, 95% CI − 0.77 to − 0.07, *I* ^2^ = 84%, *p* = < 0.01) and childbirth (*n* = 4, SMD − 1.48, 95% CI − 2.19 to − 0.76, *I* ^2^ = 93%, *p* = < 0.01). However, the effect was not significant for dressing changes (*n* = 2, SMD − 0.17, 95% CI − 0.49 to 0.15, *I* ^2^ = 0%, *p* = 0.56) (Additional File 1: Figure S6). The reduction in anxiety was significant across parallel-group trials (*n* = 13, SMD − 0.85, 95% CI − 1.14 to − 0.56, *I* ^2^ = 91%, *p* = < 0.01) but not in crossover trials (*n* = 2, SMD − 0.31, 95% CI − 0.80 to 0.17, *I* ^2^ = 38%, *p* = 0.20) (Additional File 1: Figure S6).

Only 46 trials reported on side effects with the use of VR technology (46/92, 59%). The far majority reporting mild side effects including nausea, vomiting, and headache. No serious side effects were reported (Additional File 1: Table S1).

## Discussion

### Summary of main findings

Our review summarised evidence sought from different medical disciplines evaluating the efficacy of VR technology. Despite heterogeneity, the reduction in pain perception was consistent across different clinical settings, medical procedures, and patient characteristics. We identified a relatively high number of relevant trials, particularly within the last 5 years. This was associated with a gradual development in the VR equipment used moving from larger head mount display screens to lighter and cheaper smartphones interfaces [100–103, 105–107, 115, 116, 118–122, 125–128, 130, 133, 155, 159, 160, 164]. The reduction in pain scores was observed across all evaluated medical procedures, participant age groups and trial designs, which increased the generalisability of our findings.

### Implications for clinical practice

The rapid progress in immersive VR technology has facilitated its evaluation within different clinical settings driven by smaller, cheaper, and more user-friendly VR equipment. VR immersion was defined as according to this point of view VR is described as ‘an advanced form of human–computer interface that allows the user to interact with and become immersed in a computer-generated environment in a naturalistic fashion’ [169].

As this technology becomes more widespread within the general population, its use within the health sector will gradually become mainstream with higher user acceptability and satisfaction [170]. Unlike other disciplines, e.g. engineering [171] and education [172], where VR use has grown organically, introducing it into healthcare requires deliberate implementation steps to ensure feasibility and patients’ safety [173]. Considering the beneficial effect observed in our meta-analysis, we argue that health policy makers should incorporate the use of VR within their pain management guidelines to enable its safe adoption [174]. This is particularly relevant for certain patient groups, such as in paediatric phlebotomy [175].

### Implications for future research

Our review is focused on evaluating VR technology in acute pain relief settings, largely using non-standardised software. Such versatile and easy-to-use technology has the potential to help chronic pain patients within the community enabled by virtual reality meditation and mindfulness techniques [176]. Similarly, developing procedure or condition-specific software could also help to maximise its analgesic effect as shown by some early experimental studies [177]. Lastly, clinical implementation pathways should consider the ideal format, frequency, and timing of using VR for medical procedures as per local feasibility.

Previous systematic reviews [20, 178–180] called for larger trials to address the perceived heterogeneity. Our trim and fill analysis suggests that larger trials are unlikely to nullify the depicted cumulative beneficial effect across the trials included in our analysis, thus offering low added value.

The majority of the included trials in our review focused on acute pain control following medical intervention. VR could be a game-changer to convert several inpatient procedures to outpatient settings, thus driving down cost, hospital stay, and in-hospital complications [120].

The reduction in pain management cost alone could offer a substantial advantage to reduce the length of hospital stay and associated costs, which was estimated at around $5.4 per patient (95% CI − 11 to 156) with VR use compared to routine care [181]. In this case, VR will prove dominant without the need for a formal cost-effectiveness study.

Most of the included trials used varied pain scales with no clear justifications, which may have led to higher heterogeneity at evidence synthesis. Adopting available standardised and validated outcome measurement tools would enable precise evidence synthesis and help to eliminate across trial heterogeneity. Leveraging the advances in VR user interfaces could enable interactive and contemporary built-in outcomes assessment, thus eliminating assessment bias in future studies.

### Strengths and limitations

The main strength of our review stems from our comprehensive approach to evaluating the efficacy of VR technology across different medical disciplines in contrast to previous reviews that focused on particular patient demographics or medical conditions [21]. We undertook a prospective registration, employed an exhaustive search strategy, and evaluated the sources of bias. We followed an established methodology to explore potential sources of heterogeneity and evaluated the risk of publication bias.

Our findings suffered some limitations, most notably the heterogeneity of effects among included trials. We explored this heterogeneity in a meta-regression which suggested a higher effect in minor procedures and in trials involving children. However, the observed beneficial effect pertaining across all evaluated subgroups with relatively narrow confidence intervals supports the overall benefit of VR technology for pain control. We explored this heterogeneity using a cumulative meta-analysis which confirmed that future trials are unlikely to change the certainty in the beneficial effect of VR in reducing pain following medical procedures. The prediction intervals also suggest that most population would see a benefit from using VR although a small portion might not observe this benefit (Additional File 1: Figure S7).

A potential source of heterogeneity could stem from the assumed variation in the reported common comparator (routine care). Several analgesic agents, doses, and frequencies could have been used in the control group across included studies which we were unable to adjust for in our analysis.

Several factors could drive this heterogeneity, including variations in the common comparator, background, type of software (e.g. interactive vs static), hardware fidelity, procedure and exposure duration, patient morbidity and pain tolerance, and measurement assessment tools. Exploring these effect modifiers is only possible using individual patient data. However, such analysis might fail to add significant value especially when evaluating a subjective outcome such as pain, even within the context of an individual patient data meta-analysis [182].

Most of the included studies had a small sample size, with some evident outliers identified on the funnel plot. To address the risk of publication bias, we conducted a cumulative and one-out trial analysis, excluding obvious outliers, which helped us to refine the effect estimates. While some of the included crossover RCTs suffered from risk of bias [124, 125, 131], our subgroup analysis supported the overall beneficial effect of VR across both crossover and parallel-group RCTs. Majority of the included crossover trials only reported on the effect estimates after the final crossover step which limited our ability to adjust for the potential risk of bias when pooling data from such trials. We explored the limitation of evidence sought from crossover trials using a subgroup analysis which demonstrated a wider confidence intervals compared to evidence from parallel-group trials. However, evidence of reduction in pain scores remained significant (Additional File 1: Figure S2).

Lastly, we were unable to report on the planned secondary outcomes in our protocol due to limitations in reporting across the included trials. 

## Conclusions

Immersive VR technology offers effective pain control across various medical procedures, albeit statistical heterogeneity, albeit statistical heterogeneity. Further research is needed to inform the safe adoption of this technology across different medical disciplines.

### Supplementary Information

**Additional file 1: Appendix S1.** Search strategy to identify relevant randomised trials evaluating the effectiveness of virtual reality for pain control in medical procedures. **Appendix S2.** List of studies excluded from the systematic review on the effectiveness of virtual reality for pain control in medical procedures. **Appendix S3.** List of studies excluded from the meta-analysis due to limited outcome reporting. **Figure S1.** Risk of bias in included randomised trials evaluating the effectiveness of virtual reality for pain control in medical procedures. **Figure S2.** Subgroup meta-analyses on the effectiveness of VR technology for pain control compared to routine care across different medical procedures. **Figure S3.** Funnel, Galbraith, and Trim and fill funnel plots evaluating risk of publication bias in randomised trials evaluating the effectiveness of VR technology for pain control compared to routine care across different medical procedures. **Figure S4.** One-out and sensitivity meta-analysis excluding outlier studies evaluating the effectiveness of VR technology for pain control compared to routine care across different medical procedures. **Figure S5.** Meta-analysis on the effectiveness of VR technology on anxiety compared to routine care across different medical procedures. **Figure S6.** Subgroup meta-analyses on the effectiveness of VR technology on anxiety compared to routine care across different medical procedures. **Figure S7.** Prediction intervals for the pooled effect size on pain reduction with the use of VR technology compared to routine care across all comparable populations. **Table S1.** Characteristics of randomised trials evaluating the effectiveness of virtual reality for pain control in medical procedures. **Table S2.** Meta-regression evaluating the impact of covariates on the effectiveness of VR technology on pain control across different covariates.

## Data Availability

The datasets used and/or analysed during the current study are available from the corresponding author on reasonable request.

## Abbreviations

RCT: Randomised clinical trials
VR: Virtual reality
