# Supplementary material for: Efficacy of virtual reality for pain relief in medical procedures: a systematic review and meta-analysis
Source: BMC Med. 2024 Feb 14;22:64. doi: 10.1186/s12916-024-03266-6 (PMC10865524; doi:10.1186/s12916-024-03266-6)
Supplement: Supplementary file 1 — Additional file 1: Appendix S1. Search strategy to identify relevant randomised trials evaluating the effectiveness of virtual reality for pain control in medical procedures. Appendix S2. List of studies excluded from the systematic review on the effectiveness of virtual reality for pain control in medical procedures. Appendix S3. List of studies excluded from the meta-analysis due to limited outcome reporting. Figure S1. Risk of bias in included randomised trials evaluating the effectiveness of virtual reality for pain control in medical procedures. Figure S2. Subgroup meta-analyses on the effectiveness of VR technology for pain control compared to routine care across different medical procedures. Figure S3. Funnel, Galbraith, and Trim and fill funnel plots evaluating risk of publication bias in randomised trials evaluating the effectiveness of VR technology for pain control compared to routine care across different medical procedures. Figure S4. One-out and sensitivity meta-analysis excluding outlier studies evaluating the effectiveness of VR technology for pain control compared to routine care across different medical procedures. Figure S5. Meta-analysis on the effectiveness of VR technology on anxiety compared to routine care across different medical procedures. Figure S6. Subgroup meta-analyses on the effectiveness of VR technology on anxiety compared to routine care across different medical procedures. Figure S7. Prediction intervals for the pooled effect size on pain reduction with the use of VR technology compared to routine care across all comparable populations. Table S1. Characteristics of randomised trials evaluating the effectiveness of virtual reality for pain control in medical procedures. Table S2. Meta-regression evaluating the impact of covariates on the effectiveness of VR technology on pain control across different covariates. [file 12916_2024_3266_MOESM1_ESM.docx]

**Additional File 1:**

**Additional File 1:**

**-Appendix S1:** Search strategy to identify relevant randomised trials evaluating the effectiveness of virtual reality for pain control in medical procedures.

**-Appendix S2:** List of studies excluded from the systematic review on the effectiveness of virtual reality for pain control in medical procedures.

**-Appendix S3:** List of studies excluded from the meta-analysis due to limited outcome reporting.

**-Figure S1:** Risk of bias in included randomised trials evaluating the effectiveness of virtual reality for pain control in medical procedures.

**-Figure S2:** Subgroup meta-analyses on the effectiveness of VR technology for pain control compared to routine care across different medical procedures.

**-Figure S3:** Funnel, Galbraith, and Trim and fill funnel plots evaluating risk of publication bias in randomised trials evaluating the effectiveness of VR technology for pain control compared to routine care across different medical procedures.

**-Figure S4:** One-out and sensitivity meta-analysis excluding outlier studies evaluating the effectiveness of VR technology for pain control compared to routine care across different medical procedures.

**-Figure S5:** Meta-analysis on the effectiveness of VR technology on anxiety compared to routine care across different medical procedures.

**-Figure S6:** Subgroup meta-analyses on the effectiveness of VR technology on anxiety compared to routine care across different medical procedures.

**-Figure S7:** Prediction intervals for the pooled effect size on pain reduction with the use of VR technology compared to routine care across all comparable populations.

**-Table S1:** Characteristics of randomised trials evaluating the effectiveness of virtual reality for pain control in medical procedures.

**-Table S2:** Meta-regression evaluating the impact of covariates on the effectiveness of VR technology on pain control across different covariates.

**Appendix S1:** Search strategy to identify relevant randomised trials evaluating the effectiveness of virtual reality for pain control in medical procedures.

1 exp Virtual Reality Exposure Therapy/

2 (virtual or virtuality or VR).mp.

3 (Computer interface or computer simulation).mp.

4 ((Simulated or augmented or mediated or mixed) adj3 (reality or world* or environment*)).mp.

5 ((Head or helmet) adj mounted).mp.

6 (Immersi* or Interact*).mp.

7 Distract*.mp.

8 1 or 2 or 3 or 4 or 5 or 6 or 7

9 exp Pain/

10 (Pain* or anesthe* or analges*).mp.

11 exp Fear/

12 (Anxious* or anxiet* or distress* or fear* or worry* or agitat* or apprehensi* or discomfort*).mp.w m

13 9 or 10 or 11 or 12

14 (RCT or Randomised trial or Randomised control trial).mp.

**Appendix S2:** List of studies excluded from the systematic review on the effectiveness of virtual reality for pain control in medical procedures.

-Non-randomised studies (113).

-Dental procedure (114-116).

-Did not report outcomes of interest (117,118).

-Insufficient data reported (119-121).

-No relevant population or intervention (122-153).

-Study protocol (154).

**Appendix S3:** List of studies excluded from the meta-analysis due to limited outcome reporting.

| Study | Reason |
| --- | --- |
| Canares 2001 | Did not report pre and post pain scores |
| Chan 2019a and Chan 2019b | Did not report objective pain score numerically. |
| Clerc 2021 | Did not report objective pain score numerically. |
| Esterlla-Juarez 2022 | No pain score reported, only anxiety scores. |
| Frey 2018 | Did not report objective pain score numerically. |
| Schlechter 2021 | Did not report objective pain score numerically. |
| Schmitt 2011 | Did not report objective pain score numerically. |
| Stunden 2021 | No pain score reported, only anxiety scores. |

**Figure S1:** Risk of bias in included randomised trials evaluating the effectiveness of virtual reality for pain control in medical procedures.

a: Parallel group RCTs


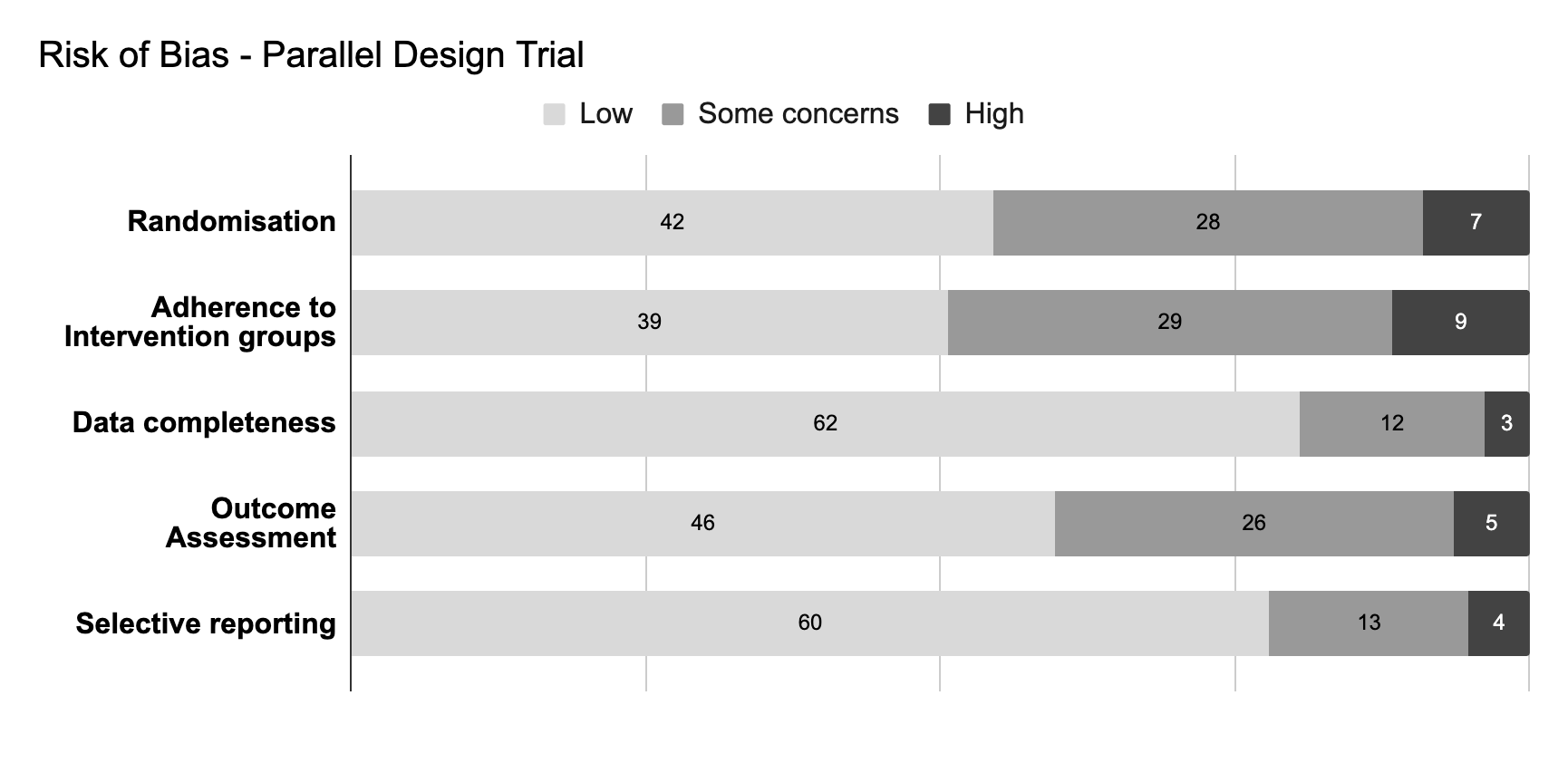


b: Crossover RCTs


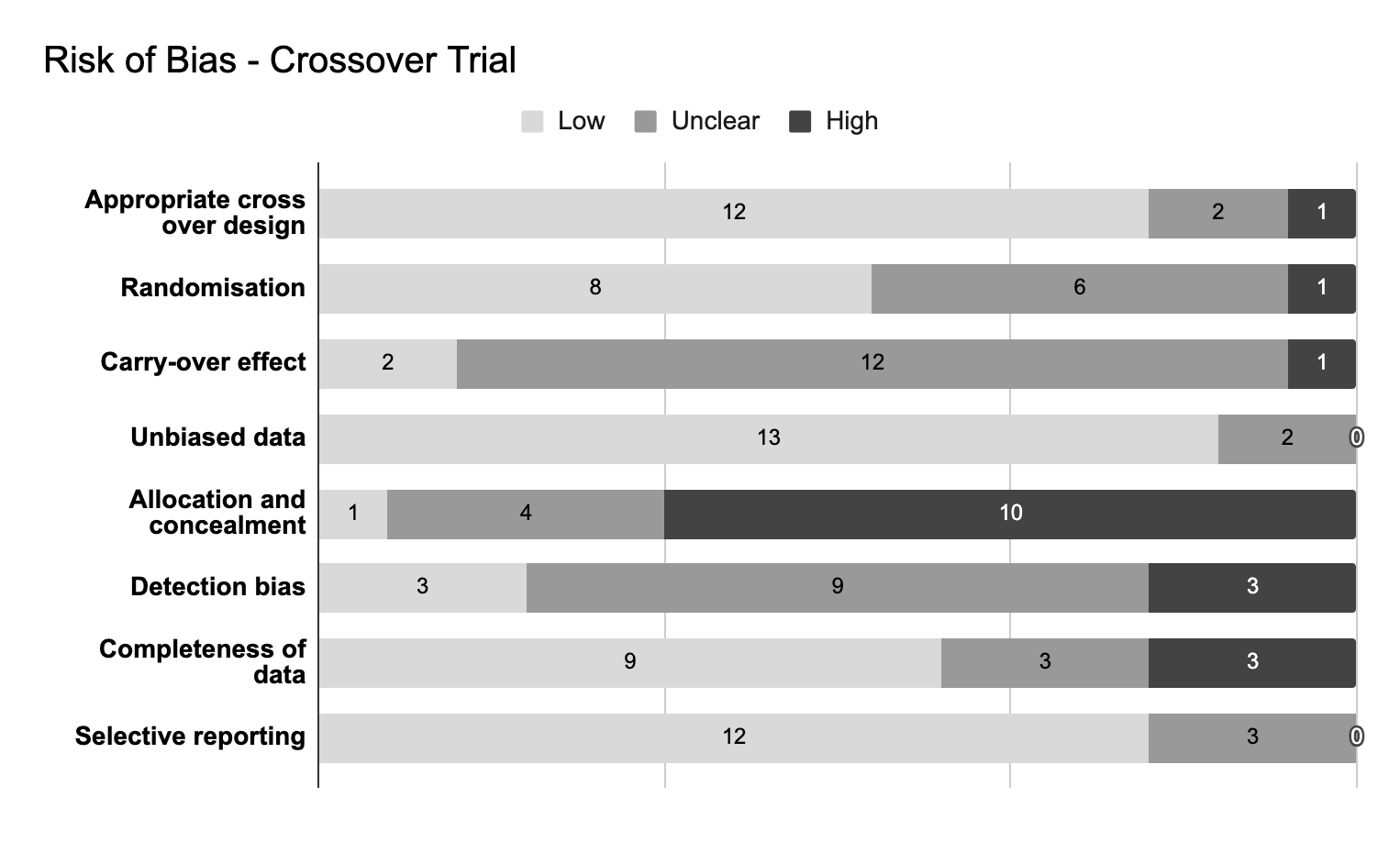


**Figure S2:** Subgroup meta-analyses on the effectiveness of VR technology for pain control compared to routine care across different medical procedures.


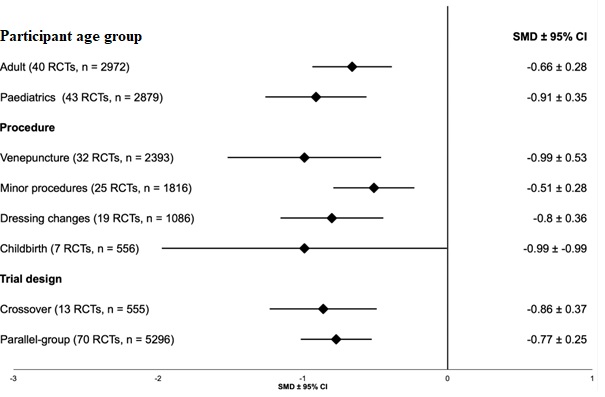


**Figure S3:** Funnel, Galbraith, and Trim and fill funnel plots evaluating risk of publication bias in randomised trials evaluating the effectiveness of VR technology for pain control compared to routine care across different medical procedures.

a: Funnel plot


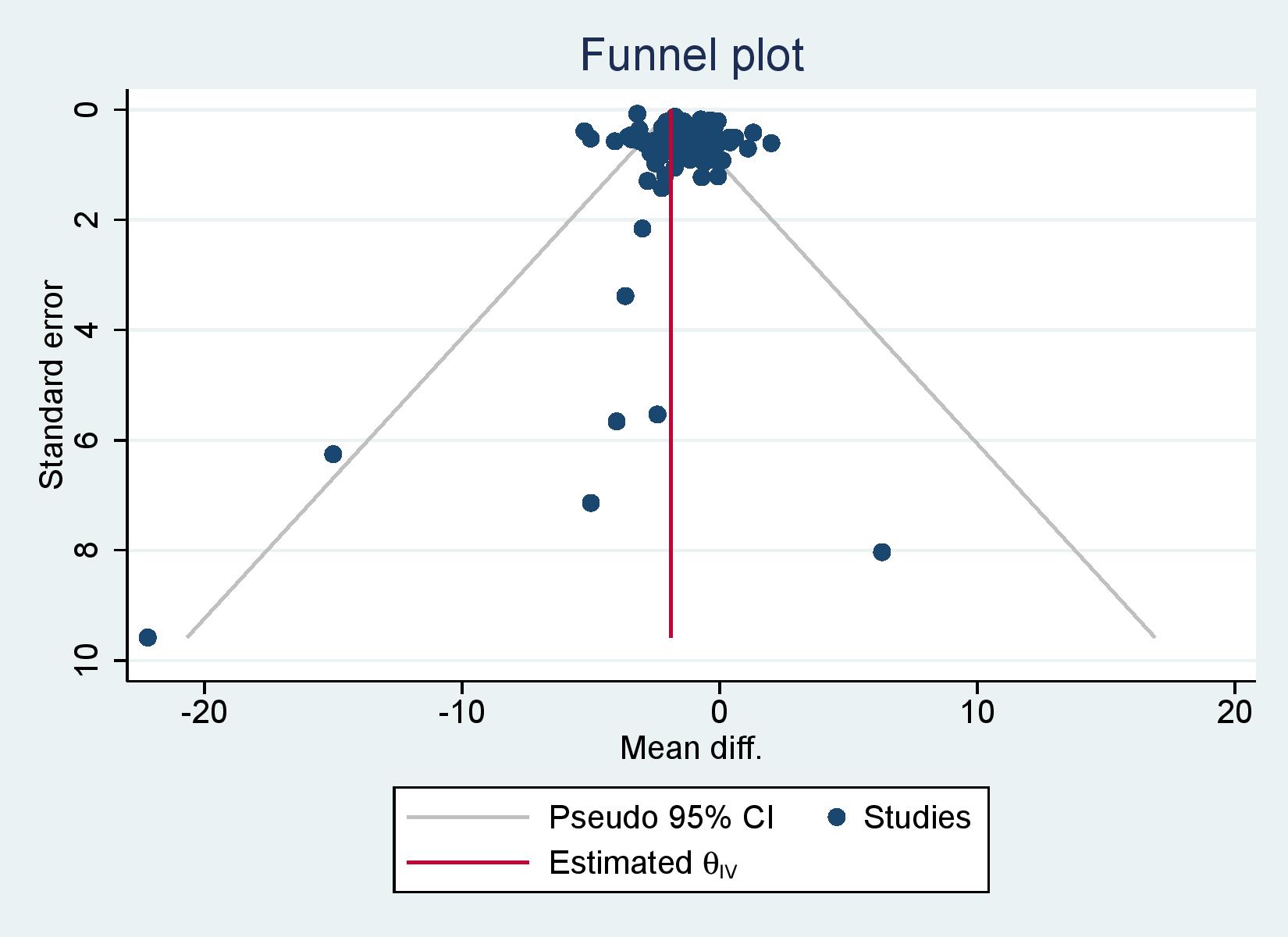


b: Galbraith plot


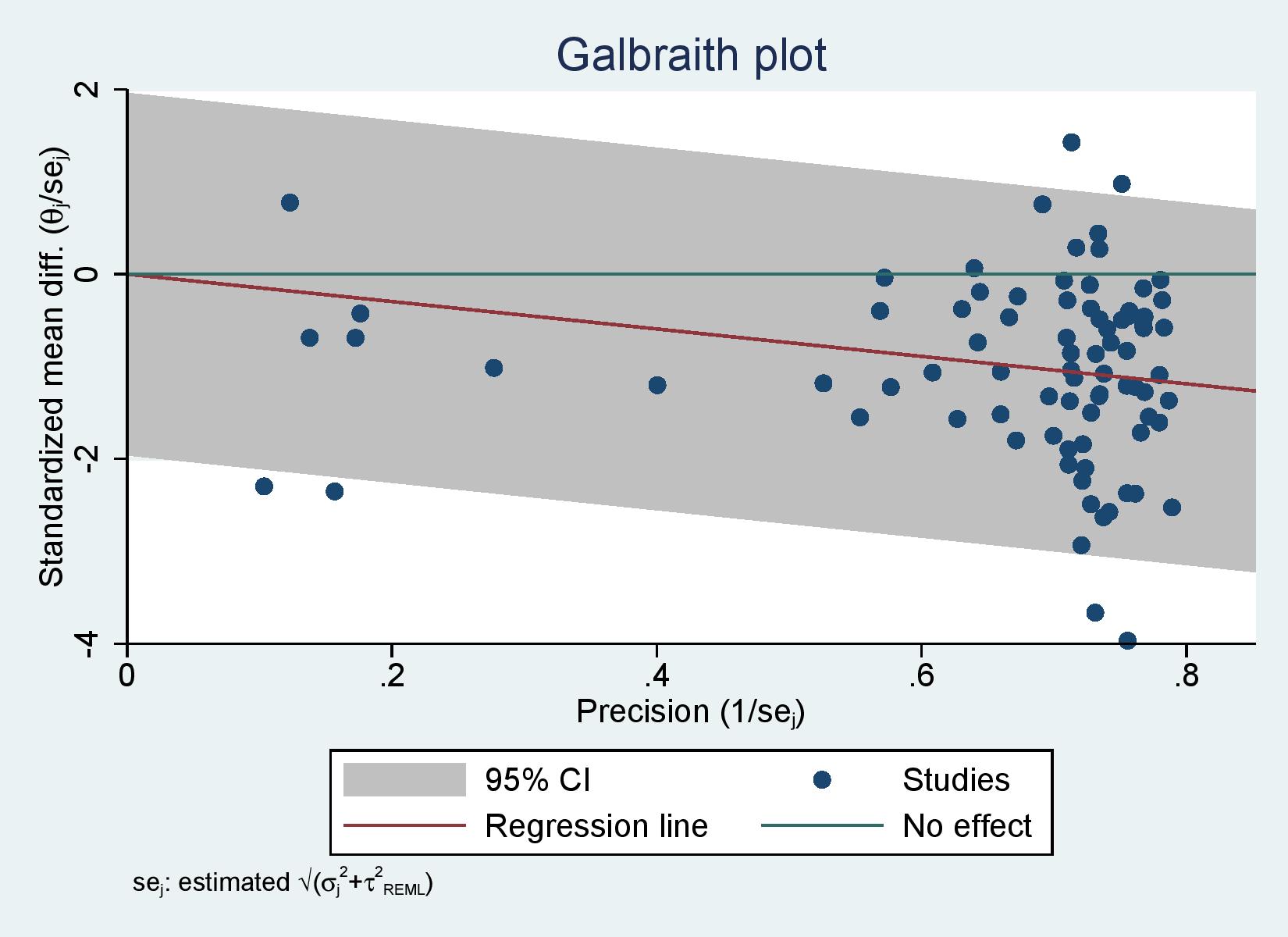


c: Trim and fill funnel plot


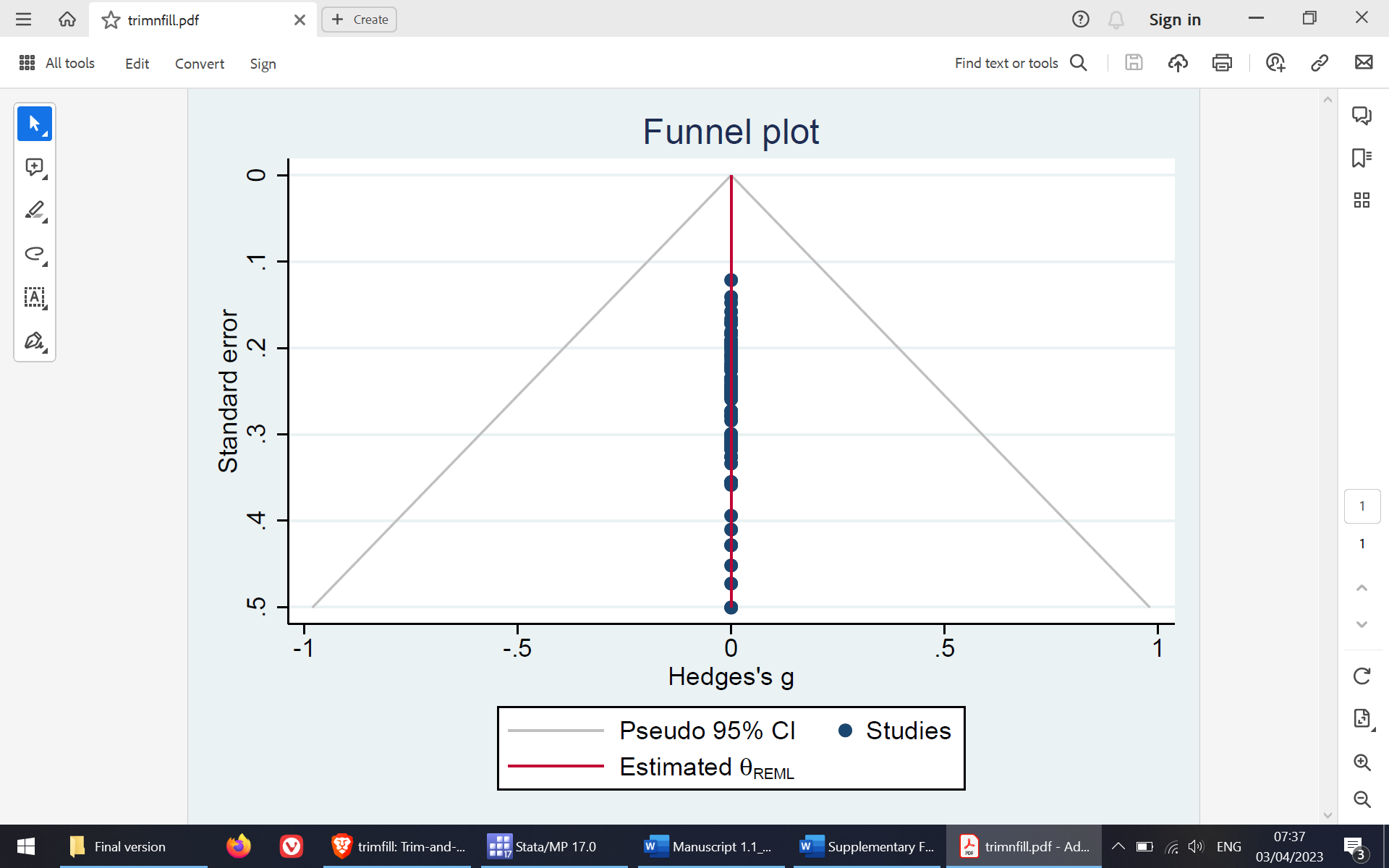


**Figure S4:** One-out and sensitivity meta-analysis excluding outlier studies evaluating the effectiveness of VR technology for pain control compared to routine care across different medical procedures.

a: One-out meta-analysis


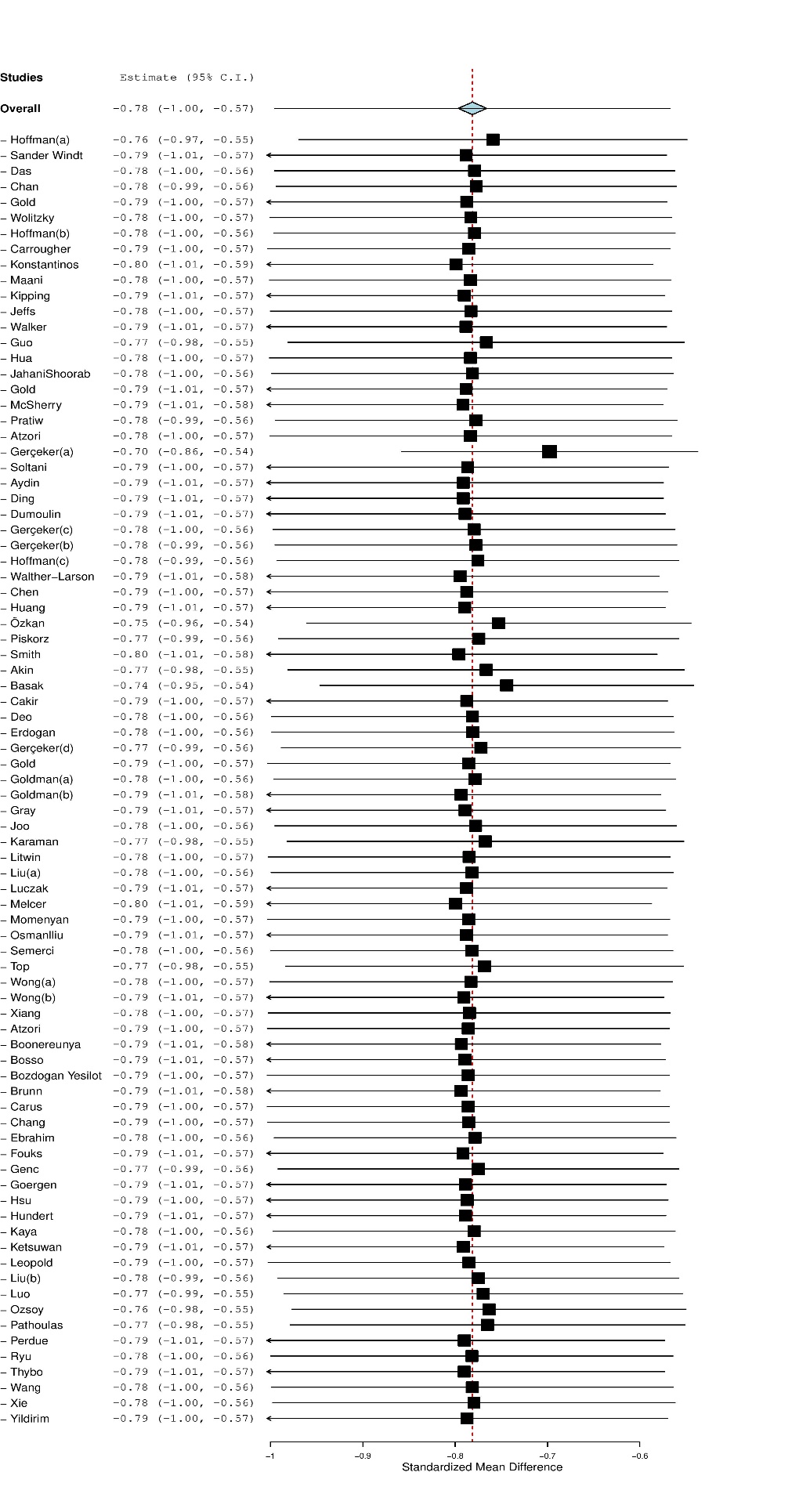


b: sensitivity meta-analysis excluding outlier studies


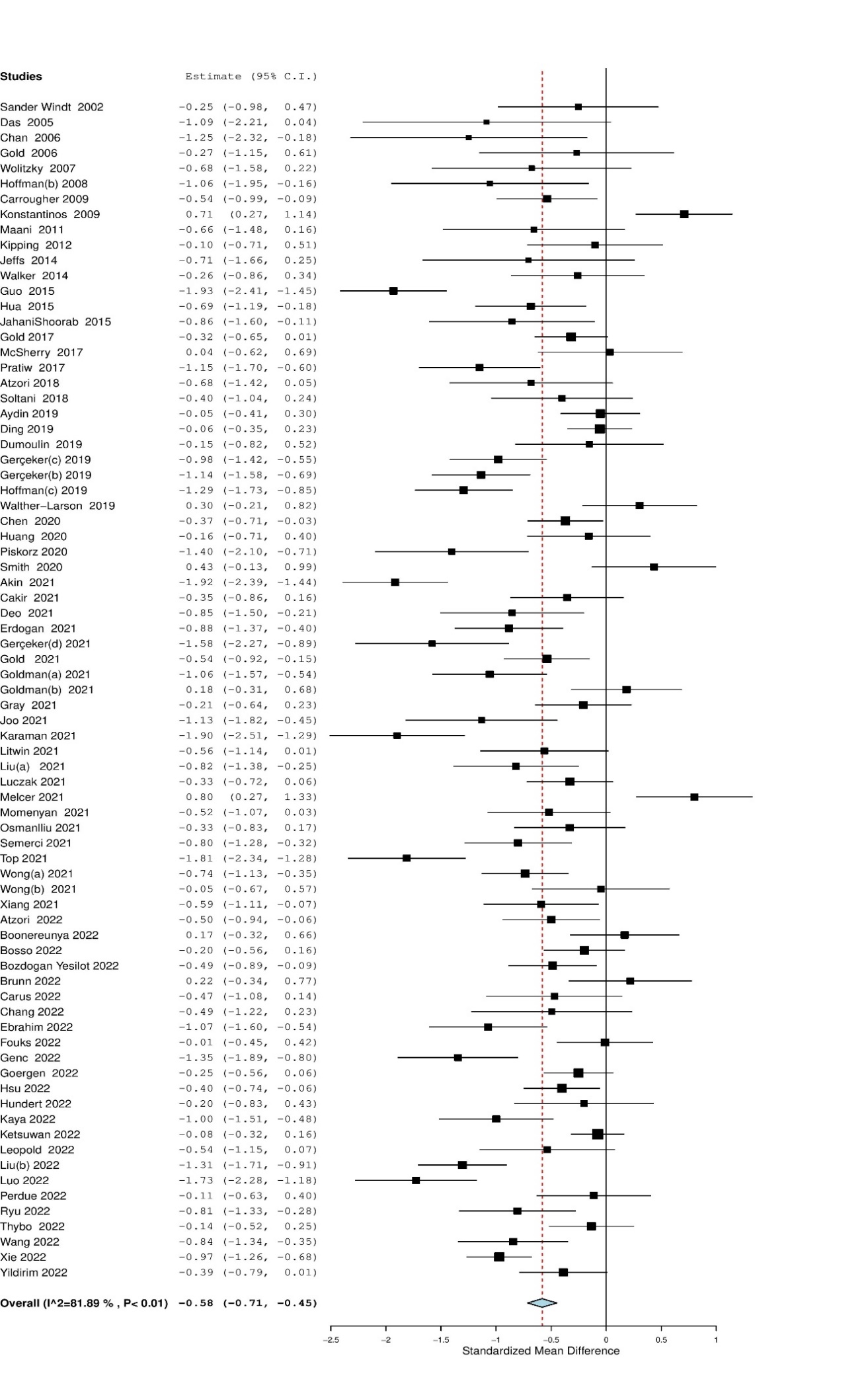


**Figure S5:** Meta-analysis on the effectiveness of VR technology on anxiety compared to routine care across different medical procedures.


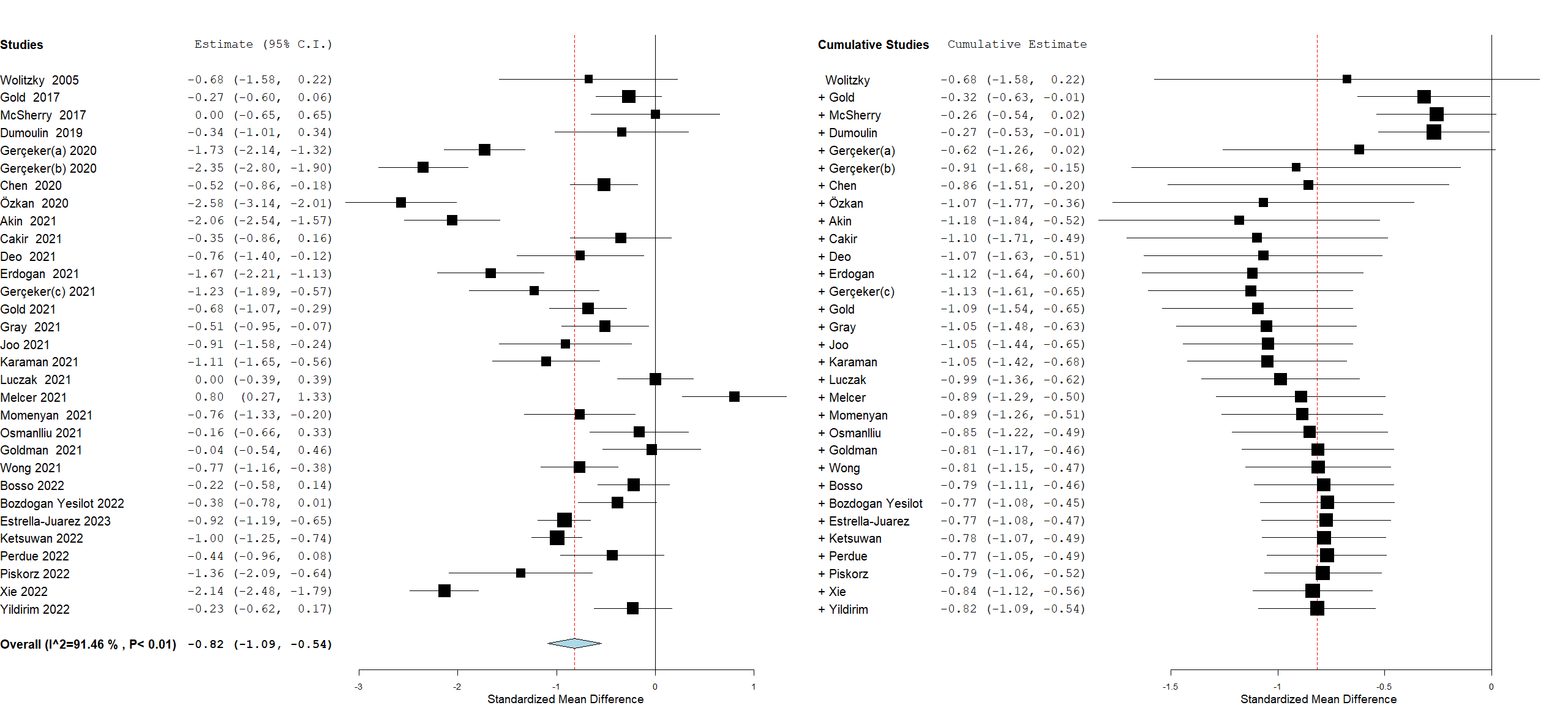


**Figure S6:** Subgroup meta-analyses on the effectiveness of VR technology on anxiety compared to routine care across different medical procedures.


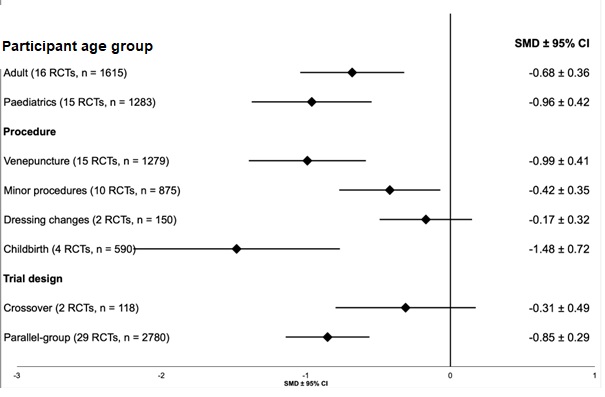


**Figure S7:** Prediction intervals for the pooled effect size on pain reduction with the use of VR technology compared to routine care across all comparable populations.

**Table S1:** Characteristics of randomised trials evaluating the effectiveness of virtual reality for pain control in medical procedures.

| Study | Country | Population characteristics | Inclusion criteria | Settings | Sample size | Medical procedure | Duration of procedure or intervention where appropriate | Pain and/or anxiety scale used | Adverse effect |
| --- | --- | --- | --- | --- | --- | --- | --- | --- | --- |
| Parallel group Trials | | | | | | | | | |
| Akin 2021 | Turkey | Women | >18 years, Primiparous, 28th week of pregnancy, cephalic presentation | IP | 100 | Labour, IP | Intervention 14.2 ± 14.9 minutes | VAS, Perinatal Anxiety Screening Scale (PASS) | No side effects reported |
| Atzori 2022 | Italy | Children | 7-17 years, with different kidney disease and understand Italian | OP | 82 | Venepuncture | Not specified | NRS | No different in mean levels of nausea |
| Aydin 2019 | Turkey | Children | 9-12 years, no developmental problems and not wearing glasses | OP | 120 | Venepuncture | Minimal (2-3 minutes) | VAS, WBFS | Not specified |
| Basak 2021 | Turkey | Adult | Native Turkish speaking, no vision and hearing problems, had a previous IM injection | ED | 91 | IM benzathine penicillin injection | Minimal (3-5 minutes) | VAS | Not specified |
| Boonreunya 2022 | Thailand | Adults | 1st experience of OGD and indication for 2% lidocaine spray pharyngeal anaesthesia | IP | 96 | OGD | 9.2 ± 2.7 minutes,  10.3 ± 3.3 (control) | VAS | All grade 1 or lower (vomit, spit, nausea), 84% no events, no significant difference in percentage reported |
| Bosso 2022 | Switzerland | Adults | ≥18 years requiring include minor procedures | ED | 117 | A&E minor procedures: suturing, wound exploration, casting, fracture reduction of joint dislocation or fracture, thoracotomy, paracentesis, or ABG measurement | Not specified | VAS | Cybersickness in 51% of patients, no patient vomited, no significant between two groups |
| Bozdogan Yesilot 2022 | Turkey | Adults | ≥ 18 years diagnosed with lipoma after superficial ultrasound | IP (elective day surgery performed in the OR) | 100 | Lipoma excision | Not specified | NRS, STAI | Not specified |
| Brunn 2022 | USA | Women | >18 years, English speaking, with no current narcotic use, indicated for hysteroscopy | OP | 50 | Hysteroscopy | Minimal (median 293 seconds (VR), median 238 seconds (control) | VAS | Not specified |
| Cakir 2021 | Turkey | Adult | >18 years, indicated for colonoscopy screening | IP | 60 | Colonoscopy | Median 7.4 min (VR), Median 7.3 min (control) | VAS, STAI | Not specified |
| Canares 2021 | USA | Children | >7 years | ED | 55 | Venipuncture or IV placement | Not specified | Child-Adult Medical Procedure Interaction Scale-Short Form (CAMPIS-SF) | No significant presence of cybersickness side effect |
| Carus 2022 | Turkey | Women | 18-42 years, 37-41 weeks gestation, cephalic presentation, for vaginal birth | IP | 42 | Labour (early and first stage) | VR of 20 minutes in two phases each | Wong-Baker Faces Pain Rating Scale, Beck Anxiety Inventory | No significant adverse events observed |
| Chan 2019a | Australia | Children | 4-11 years, required IV access for any indication | ED | 123 | Venepuncture & IV placement | Minimal | Child-rated Faces Pain Scale-Revised (FPS-R) | No adverse effect in VR group |
| Chan 2019b | Australia | Children | 4-11 years, required IV access for any indication | OP | 129 | Venepuncture | Minimal | Child-rated FPS-R | Rare and minor, 3 had nausea, headache (3 in control) |
| Chang 2022 | Singapore | Children | 4-10 years accompanied by parents or legal guardians | Primary care | 30 | Paediatric immunisation | 2 minutes | FPS-R, VAS | No adverse effect reported |
| Chen 2020 | Taiwan | Children | 7-12 years, conscious, Mandarin or Taiwanese speaking, | IP | 136 | IV injection | 53.5 ± 19.0 seconds,  61.3 ± 25.8 (control) | Wong-Baker Faces Scale, Children’s Fear Scale | Not specified |
| Clerc 2021 | Canada | Children | 6-16 years, for minor plastic procedure | IP | 64 | Minor paediatric plastic surgery (simple excisions, serial excisions, biopsies, scar revisions, partial wound closures, steroid injections, trigger finger releases, and other clinic-based procedures that could be performed under local anaesthesia) | 22 (IQR 20-29) minutes,  29 (23-37)(control) | FPS-R and Venham situational anxiety scale | Two reported dizziness and one reported mild nausea - resolved with removal of headset |
| Deo 2021 | United Kingdom | Women | 18-70 years with no hearing or visual impairment | OP | 40 | Hysteroscopy | 3.25 minutes | NRS | Well tolerated with no serious side effects (nausea reported by 1 patient) |
| Ding 2019 | China | Adults | >18 years, Chinese speaking, with expected stay >7 days, with postoperative wounds that required daily care and dressing changes | IP | 182 | Dressing change post haemorrhoid surgery | 21.2 ± 3.8 minutes,  20.4 ± 4.1 (control) | VAS |  |
| Dumoulin 2019 | Canada | Children | 8-17 years, visiting ED for pending or known diagnostic | ED | 59 | Venepuncture & IV placement | Minimal (<15 minutes) | VAS | No significant difference in change in estimated cybersickness |
| Ebrahimian 2022 | Iran | Women | 18-35 years, 37-41 weeks gestation singleton, cephalic presentation, low risk pregnancy, Gravide 1 or 2, | IP | 93 | Labour, first stage | Intervention for 30 minutes at two time points (4-5cm and 7-8cm dilated) | Visual analogue, Spielberger’s anxiety inventory | Not specified |
| Erdogan 2021 | Turkey | Children | 7-12 years, healthy, requiring blood test | OP | 108 | Venepuncture | 3 minutes | VAS, WBFS | Not specified |
| Estrella-Juarez 2023 | Spain | Women | ≥37 weeks’ gestation, low-risk pregnancy, nulliparous, singleton, spontaneous conception | IP | 343 | Non-stress test in third trimester and first stage of labour | Intervention for 20 minutes twice | STAI | Not specified |
| Fouks 2022 | Israel | Women | ≥ 18 years, not having suspected infection, not using analgesia 6 hours prior to procedure | OP | 102 | Hysteroscopy | 8.1 ± 3.2 minutes,  7.3 ±6 6.0 (control) | NRS | No differences in dizziness, nausea, vomiting, shivering and contraction or menstrual pain |
| Genc 2022 | Turkey | Adults | >18 years, 1st biopsy, no psychiatric disease, normal PSA level | OP | 96 | Transrectal Prostate Biopsy | 10 minutes | VAS | Not specified |
| Gerçeker 2018 | Turkey | Children | 7-12 years with no mental or language deficiencies | OP | 121 | Venepuncture | Not specified | WBFS | Not specified |
| Gerçeker 2020a | Turkey | Children | 5-12 years | OP | 136 | Venepuncture | Not specified | WBFS, Children’s Anxiety metre | Not specified |
| Gerçeker 2020b | Turkey | Children | 5-12 years | OP | 136 | Venepuncture | Not specified | WBFS, Children’s Anxiety metre | Not specified |
| Gerçeker 2021 | Turkey | Children | 6-17 years, for routine chemotherapy, no previous known severe needle insertion phobia | IP | 42 | Access to venous port with Huber needle | Not specified | WBFS, Children’s Anxiety metre | No adverse events reported |
| Goergen 2022 | Brazil | Adults | ≥18 years, elective rigid cystoscopy under local anaesthesia | OP | 159 | Rigid cystoscopy - diagnostic, follow-up or for double-J stent extraction | 5.3 ± 3.2 minutes,  8.7 ± 5.0 (control) | VAS | No adverse events reported |
| Gold 2006 | USA | Children | 8-12 years, awaiting MRI or CT scans requiring placement | OP | 20 | IV placement | Not specified | FPS-R, WBFS | No simulator sickness reported |
| Gold 2017 | USA | Children | 10-21 years, English or Spanish speaking, no cognitive or developmental delay, not taking analgesia or anxiolytics | OP | 143 | Venepuncture | Not specified | VAS | 4 patients (5%) reported mild-moderate nausea, no other adverse events reported |
| Gold 2021 | USA | Children | 10-21 years, undergo peripheral placement, English or Spanish speaking, not taking analgesia or anxiolytics | OP | 107 | IV placement | Not specified | FPS-R, VAS | Not specified |
| Goldman 2021a | Canada | Children | 6-16 years, non-facial laceration that had to be repaired with suturing | ED | 62 | Laceration repair | 27.4± 19.2 minutes | FPS-R, Venham Situational Anxiety scale | Not specified |
| Goldman 2021b | Canada | Children | 6-16 years, without the highest acuity category, no trauma to face | ED | 66 | IV placement | Not specified | FPS-R, Venham Situational Anxiety scale | Not specified |
| Guo 2015 | China | Adults | ≥18 years, serious hand injuries, including hand skin avulsion,  soft tissue defects, damage to the nail bed, fingers, hands etc. caused by crush injuries or firearm injuries, debridement or suturing within 72 hours of injury | OP | 98 | Dressing change hand injury | Not specified | VAS | Not specified |
| Hsu 2022 | Taiwan | Children | 6-12 years, recommended to receive IV placement | IP | 134 | Peripheral IV line insertion | 52 ± 22 seconds,  51 ± 16 (control) | WBFS | No adverse events reported |
| Hua 2015 | China | Children | 4-16 years, with chronic wounds on lower limbs that require active dressing changes | IP | 65 | Dressing change chronic wound | 28 ± 7 minutes,  22 ± 8 (control) | WBFS | Not specified |
| Huang 2020 | Australia | Adults | ≥18 years, with no significant cardiovascular or respiratory disease, able to receive spinal block | IP | 50 | Knee or hip arthroscopy | 120 (IQR 105-140) minutes,  130 (115-140) control | 1-5 scale | 1 patient report worsening nausea present prior to procedure, no other adverse outcomes reported |
| JahaniShoorab 2015 | Iran | Adults | Low risk pregnancy without obstetric complication, no history of mental illness, addiction, motion sickness and headaches | IP | 30 | Episiotomy repair | 11.4 ± 2.6 minutes,  13.6 ± 3.3 (control) | NRS | Not specified |
| Jeffs 2014 | USA | Children | 10-17 years, English speaking, undergoing burn wound care as first-time visit to clinic | OP | 28 | Burn wound care | 32 ± 31 minutes,  49 ± 27 (control) | World GRS | None reported nausea or light-headness |
| Joo 2021 | Korea | Adults | 20-85 years, at least 3-month duration of chronic pain | OP | 38 | Lumbar sympathetic ganglion block | 6.2 ± 1.3 minutes,  6.1 ± 1.2 (control) | NRS | 2 reported transient dizziness, 3 reported discomfort (no dropouts), no other adverse events reported |
| Karaman 2021 | Turkey | Women | ≥18 years with palpable breast masses | OP | 60 | FNA breast biopsies | 5-6 minutes on average | VAS, State-Trait Anxiety Inventory | Not specified |
| Kaya 2022 | Turkey | Children | 7-12 years, first attendance, 2nd degree superficial and deep burn, <10% burns | IP | 65 | Dressing change burn patients | Not specified | Wong-Baker Faces Pain Rating Scale, Child STAI | Not specified |
| Ketsuwan 2022 | Thailand | Adults | ≥18 years, first attendance, no absolute contraindication for flexible cystoscopy | OP | 270 | Flexible cystoscopy | Not specified | VAS, STAI | Not specified |
| Kipping 2012 | Australia | Children | 11-18 years, first conscious change of dressing, burn wound TBSA >1% | IP and OP | 41 | Burn wound care | 10 minutes (IQR 4-20), 12 (9-20)(control) | VAS | No adverse events reported |
| Konstantinos 2009 | Australia | Adults | 18-80 years, burns requiring admission and able to use PCA opioid | IP | 86 | Dressing change burn patients | 77 ± 35 minutes,  77 ± 27 (control) | VAS | Not specified |
| Leopold 2022 | USA | Adult |  | IP | 43 | Interventional radiology procedure (Thyroid biopsy, minor venous access procedures) | Not specified | VAS |  |
| Litwin 2021 | Canada | Children | 8-17 years requiring IV insertion, English speaking, deemed medically stable | ED | 48 | IV placement | 4 ± 3.7 minutes,  4.1 ± 4 (control) | NRS | No safety concerns or significant adverse effects |
| Liu 2021 | USA | Children | 7-17 years indicated for nasal endoscopy | OP | 53 | Flexible or rigid nasal endoscopy | 80 ± 23 seconds,  82 ± 26 (control) | WBFS, Subjective units of distress (SUDS) anxiety score | No adverse events reported |
| Liu 2022 | China | Adults | 20-75 years, sedation-free colonoscopy, not taking beta blockers or buscopan | OP | 117 | Colonoscopy | Median 15 minutes,  16 minutes (control) | VAS | Not specified |
| Luczak 2021 | Poland | Adult | Men and women, Indicated due to primary or follow-up diagnostics | OP | 103 | Rigid cystoscopy | Not specified | NRS | Few patient reported moderate vertigo or nausea (none procedures were interrupted) |
| Luo 2022 | China | Children | 7-18 years with pathological phimosis, or recurrent balanitis or foreskin or balanitis xerotica obliterans | IP | 70 | Circumcision | Not specified | VAS, FPS-R | No major adverse events reported, four in total (motion sickness or dizziness) |
| Melcer 2021 | Israel | Women | Singleton pregnancy, Obstetrically indicated mid-trimester amniocentesis | OP | 60 | Amniocentesis | Not specified | VAS | Well tolerated with no serious side effects, 2 removed headset due to nausea |
| Momenyan 2021 | Iran | Women | 18-45 years, >38 weeks gestation, low risk pregnancy, vaginal delivery, no other methods of analgesia | IP | 52 | Labour (first and second stage) | 10 minute intervention | NRS | Not specified |
| Osmanlliu 2021 | Canada | Children | 7-17 years requiring IV procedure, French or English speaking | IP | 62 | Venepuncture & IV placement | Not specified | VerbalNRS | No serious adverse event reported. Mild side effects: dizziness (3), nausea (1), headache (1) |
| Özkan 2020 | Turkey | Children | 4-10 years receiving a routine health check up | IP | 135 | Venepuncture | Not specified | VAS, WBFS | Not specified |
| Ozsoy 2022 | Turkey | Children | 7-10 years, at least 2 days since operation, having undergone appendectomy or small abdominal surgery, Turkish speaking | IP | 64 | Dressing change | Not specified | WBFS | Not specified |
| Perdue 2022 | USA | Adults | 18-50 years, had orders to undergo blood testing | OP | 58 | Venepuncture | Not specified | VAS | No adverse effects reported |
| Piskorz 2020 | Poland | Children | 7-17 years, patients at paediatric nephrology clinic suffering for kidney failure | IP | 57 | Port site access | Not specified | 1-10 pain scale | Not specified |
| Pratiw 2017 | Indonesia | Adults | >36 weeks gestation, Indonesian national, low risk pregnancy | IP | 60 | Labour (latent phase and active phase of first stage) | 3 interventions lasting 10 minutes each (latent phase, 4-5cm, 7-8cm) | Faces Pain Scale, Nonverbal Pain Scale, | Not specified |
| Ryu 2022 | Korea | Children | 4-8 years, no cognitive deficits, no prior experience of venepuncture during past year | IP | 60 | Venepuncture | 52 (IQR 36-67) seconds, 51 (IQR 43-72)(control) | VAS, Children’s Hospital of Eastern Ontario Pain Scale | Not specified |
| Sander Windt 2002 | USA | Children | 10-19 years, being treated for cancer, undergoing at least a second LP, English speaking | IP | 30 | Lumbar puncture | Not specified | VAS | Not specified |
| Schlechter 2021 | USA | Children | 4-17 years requiring IV placement as part of ED care | ED | 116 | IV placement | Minimal | FPS-R, Likert-type anxiety scale | No serious adverse event report, no emesis |
| Semerci 2021 | Turkey | Children | 7-17 years requiring venous port access in paediatric oncology | IP | 71 | Port-a-cath access | Not specified | WBFS | Not specified |
| Smith 2020 | Australia | Adults | Singleton pregnancy, USS confirmed breech presentation | IP | 50 | External cephalic version | 623 ± 722 seconds,  439 ± 752 (control) | 101 point numerical rating scale | 26% experienced side effect, no difference between two groups: dizziness, nausea, vomiting, tremulousness, flushing |
| Soltani 2018 | USA | Mixed | 15-18 years, requiring therapies for ROM exercise with ability to perform independently | IP | 39 | Range of movement exercise (ROM) in burn patients | 2.8 ± 1.7 minutes,  2.8 ± 1.6(control) | GRS | Not specified |
| Stunden 2021 | USA | Children | 4-13 years, English speaking | IP | 92 | Paediatric MRI | 22.1 ± 4.4 minutes,  15.1 ± 3.3 (control) | Venham picture test (VPT) | 1 expressed eye strain, no other side effect reported |
| Thybo 2022 | Denmark | Children | 4-7 years, Danish Speaking, admitted for preoperative venous cannulation | IP | 106 | IV placement | 120 (IQR 60-165) seconds,  110 (60-180)(control) | WBFS | Few adverse effects, no significant differences between two groups |
| Top 2021 | Turkey | Children | 4-6 years admitted to hospital | IP | 80 | Venepuncture | Mean 4 minutes | FPS-R | Not specified |
| Walker 2014 | USA | Adults | 18-70 years, English speaking, male, first procedure | OP | 43 | Flexible cystoscopy | Not specified | VAS | No adverse events reported to include nausea or dizziness |
| Walther−Larson 2019 | Denmark | Children | 7-16 years, Danish speaking, before induction of anaesthesia | IP | 59 | IV placement | 1.8 (IQR 1-3.6) minutes, 2 (IQR 1-2.5)(control) | VAS | Few adverse effects (nausea, dizziness, other discomfort), no significant difference between 2 groups |
| Wolitzky 2005 | USA | Children | 7-14 years, receiving treatment for cancer | IP | 20 | Port access | Unspecified | VAS | Not specified |
| Wong 2021a | Hong Kong | Children | 6-17 years, Chinese speaking, diagnosed with cancer | IP | 108 | IV placement | 2.7 ± 0.7 minutes,  3.4 ± 2.1 (control) | VAS, State Anxiety Scale | No adverse effects reported |
| Wong 2021b | USA | Adults | ≥18 years, English speaking, nulliparous, term, having contractions at least every 5 minutes for preceding 30 minutes, pain score of 4-7 on VAS | IP | 40 | Labour, first stage | 30 minutes of intervention | VAS | 1 adverse event: emesis after 16 minutes of use, patient discontinued |
| Xiang 2021 | USA | Children | 6-17 years with burn injury, English speaking | OP | 90 | Dressing change burn patients | <30 minutes, but not specified | VAS | No difference in simulator sickness score |
| Xie 2022 | China | Women | 20-34 years, singleton, no complications in pregnancy and delivery | IP | 200 | Labour, second stage | 95 ± 10 minutes,  96 ± 12 (control) | VAS, VAS-A | Not specified |
| Yildirim 2023 | Turkey | Children | 4-10 years, being conscious | ED | 150 | IV placement | 10.5 minutes,  9.9 minutes (control) | WBFS, Children’s Anxiety Meter-State | Not specified |
| Crossover Trials | | | | | | | | | |
| Atzori 2018 | Italy | Children | 7-17 years, Italian speaking | OP | 15 | Venepuncture and IV placement | Not specified | VAS | No adverse effect reported |
| Carrougher 2009 | USA | Adults | >20 years, English speaking, requiring postburn injury physical therapy on two consecutive days during their stay | IP | 39 | Range of movement exercise in burn patients | 10 minutes | GRS | Not specified |
| Chan 2007 | Taiwan | Children | Experienced burns for first time, range from 1st-4th degree burns | IP | 8 | Dressing change burn patients | 15-20 minutes | Faces scale (0-100) | Not specified |
| Das 2005 | Australia | Children | 5-18 years, having burns more than 3% of body surface area, requiring dressing changes | IP | 9 | Dressing change burn patients | Not specified | Faces Scale | Not specified |
| Frey 2018 | USA | Adults | Healthy, >32 weeks’ gestation, nulliparous, vaginal delivery, low risk pregnancy | IP | 27 | Labour, first stage | Unspecified | NRS | No adverse effect, no significant differences in nausea |
| Gray 2021 | USA | Adults | 18-65 years requiring post-operative nasal endoscopy and debridement after undergoing functional endoscopic sinus surgery or endoscopic endonasal skull base surgery | OP | 82 | Nasal endoscopy and debridement | 329 ± 199 seconds,  370 ± 243 (control) | VAS, Subjective units of distress scale (SUDS) | No adverse effects reported |
| Hoffman 2001 | USA | Mixed | 9-32 years, 3-60% total body surface area burned, reported previous trouble tolerating pain during physical therapy | IP | 7 | Range of movement exercise in burn patients | Between 3.5 to 9 minutes | VAS | Not specified |
| Hoffman 2008 | USA | Mixed | 9-40 years with burns severe enough for admission | IP | 11 | Burn wound debridement | 3 minutes with VR and 3 minutes without | GRS | Not specified |
| Hoffman 2019 | USA | Children | 6-17 years, >10% burns total body surface area, English or Spanish speaking | IP | 48 | Burn wound cleaning | Alternating between VR and no VR for 5 minutes | GRS | Nausea reported nearly zero (<1) |
| Hundert 2022 | Canada | Children | 8-18 years, English speaking, actively undergoing treatment for cancer, 1 month-3 years from initial diagnosis, requiring at least 2 insertions for cancer-related treatment over 8 weeks | OP | 40 | Subcutaneous port access | Not specified | NRS | No serious adverse events or harms reported, no difference in dizziness or motion sickness |
| Maani 2011 | USA | Adults | ≥18 years with thermal injuries, can operate a computer mouse or keyboard | IP | 12 | Burn wound debridement | Mean treatment segment 5.7 minutes (range 3-11 minutes) | GRS | Rate nausea during VR as zero |
| McSherry 2017 | USA | Adults | Adult patients undergoing painful wound care procedures for deep or partial thickness burns of ≥5% of complex nonburn wound, prior completion of at least 2 prior painful wound care procedure | IP | 18 | Burn wound care | 30 ± 13 minutes,  31 ± 15 (control) | Verbal numeric scale (VNS) | Not specified |
| Pathoulas 2022 | USA | Adults | Undergoing treatment for platelet-rich plasma, had at least 1 prior scalp injection | IP | 18 | Scalp injections | Not specified | VAS | No adverse events occurred |
| Schmitt 2011 | USA | Children | <19 years who required post-burn, active-assisted ROM physical therapy at least once | IP | 54 | Physical therapy in burn patients | 6-20 minutes | Graphic rating scale (GRS) | Nausea rating not obtained from controls |
| Wang 2022 | China | Children | 8-14 years with mild burns, 2nd-3rd degree burns, <10% burn area | OP | 34 | Dressing change burn patients | 9.2 ± 6.5 minutes,  8.1 ± 4.2 (control) | WBFS | Mild nausea reported (but no percentages) |

* FPS-R, Faces Pain Scale-Revised; GRS, Graphic Rating Scale; NRS, Numeric Rating Scale; STAI, Spielberger State-Trait Anxiety Inventory; VAS, Visual Analog Scale, WBFS, Wong-Baker Faces Pain Rating scale

**Table S2:** Meta-regression evaluating the impact of covariates on the effectiveness of VR technology on pain control across different covariates.

| Covariate | Coefficients | Lower bound | Upper bound | p-Value |
| --- | --- | --- | --- | --- |
| Paediatric participants | -0.237 | -0.658 | 0.184 | 0.270 |
| Crossover trials | -0.194 | -0.794 | 0.407 | 0.527 |
| Venepuncture | -0.043 | -0.826 | 0.741 | 0.915 |
| Minimally invasive medical procedures | 0.370 | -0.433 | 1.172 | 0.367 |
| Dressing changes in burn patients | 0.050 | -0.786 | 0.887 | 0.907 |
